# Epidemiological characteristics and determinants of dengue transmission during epidemic and non-epidemic years in Fortaleza, Brazil: 2011-2015

**DOI:** 10.1371/journal.pntd.0006990

**Published:** 2018-12-03

**Authors:** Benjamin MacCormack-Gelles, Antonio S. Lima Neto, Geziel S. Sousa, Osmar J. Nascimento, Marcia M. T. Machado, Mary E. Wilson, Marcia C. Castro

**Affiliations:** 1 Department of Global Health and Population, Harvard T.H. Chan School of Public Health, Boston, Massachusetts, United States of America; 2 Fortaleza Municipal Health Secretariat (SMS-Fortaleza), Fortaleza, Ceará, Brazil; 3 University of Fortaleza (UNIFOR), Fortaleza, Ceará, Brazil; 4 Federal University of Ceará (UFC), Fortaleza, Ceará, Brazil; 5 School of Medicine, University of California, San Francisco, California, United States of America; University of California, Davis, UNITED STATES

## Abstract

**Background:**

After being eliminated during the 1950s, dengue reemerged in Brazil in the 1980s. Since then, incidence of the disease has increased, as serotypes move within and between cities. The co-circulation of multiple serotypes contributes to cycles of epidemic and interepidemic years, and a seasonal pattern of transmission is observed annually. Little is known regarding possible differences in the epidemiology of dengue under epidemic and interepidemic scenarios. This study addresses this gap and aims to assess the epidemiological characteristics and determinants of epidemic and interepidemic dengue transmission, utilizing data from the 5^th^ largest city in Brazil (Fortaleza), at fine spatial and temporal scales.

**Methods/Principal findings:**

Longitudinal models of monthly rates of confirmed dengue cases were used to estimate the differential contribution of contextual factors to dengue transmission in Fortaleza between 2011 and 2015. Models were stratified by annual climatological schedules and periods of interepidemic and epidemic transmission, controlling for social, economic, structural, entomological, and environmental factors. Results revealed distinct seasonal patterns between interepidemic and epidemic years, with persistent transmission after June in interepidemic years. Dengue was strongly associated with violence across strata, and with poverty and irregular garbage collection during periods of low transmission, but not with other indicators of public service provision or structural deprivation. Scrapyards and sites associated with tire storage were linked to incidence differentially between seasons, with the strongest associations during transitional precipitation periods. Hierarchical clustering analysis suggests that the dengue burden concentrates in the southern periphery of the city, particularly during periods of minimal transmission.

**Conclusions/Significance:**

Our findings have direct programmatic implications. Vector control operations must be sustained after June even in non-epidemic years. More specifically, scrapyards and sites associated with tires (strongly associated with incidence during periods of minimal transmission), require sustained entomological surveillance, particularly during interepidemic intervals and in the urban periphery. Intersectoral collaborations that address urban violence are critical for facilitating the regular activities of vector control agents.

## Author summary

Almost half of the world population is at risk of a dengue infection. Although dengue often presents annual transmission seasonality (peaking during the summer) and features epidemics occuring between multi-year interepidemic intervals, little is known about how the epidemiology and determinants of dengue vary seasonally and during different stages of the epidemic cycle. As a result, control efforts likely neglect factors that sustain transmission between epidemics, or that set epidemics in motion. Using five years of monthly data at fine spatial scale from the 5^th^ largest city in Brazil, we observed that dengue epidemiology in interepidemic years deviates from the common pattern, showing a much longer and sustained transmission season. Occurrence of violence was a strong and consistent predictor of high dengue incidence, while specific areas in the city (e.g. with a high concentration of scrapyards and locations used for tire storage) were critical during transitional precipitation periods. Indicators of social deprivation were only correlated with dengue incidence during periods of low transmission, suggesting that practices typifying these areas–such as water storage–may extend viremic circulation during dry seasons. These findings can be translated into revised and better strategies of control, but also call for the need to better understand how variations in the epidemiology of dengue may affect the success of current control recommendations.

## Introduction

Dengue virus (DENV, genus *Flavivirus*, family *Flaviviridae*) is an arbovirus with at least four distinct serotypes (DENV1, DENV2, DENV3, and DENV4) that confer homologous immunity [1]. Manifestations of DENV infection range from completely asymptomatic in as many as three quarters of cases, to dengue hemorrhagic fever (DHF) and dengue shock-syndrome (DSS), with an intervening continuum of febrile symptoms and nonspecific signs of infection such as headache and rash [2]. Dengue occurs primarily in tropical and sub-tropical latitudes with an estimated burden of 390 million cases annually, of which 96 million cases manifest symptomatically [3]. These figures represent a thirty-fold increase in disease globally over the last fifty years that exposes nearly three billion people to the risk of infection [4]. The heaviest burden is in Asia, with an estimated 67 million symptomatic cases annually, followed by the Americas, with 13 million, of which more than half occur in Brazil and Mexico [3].

Dengue is transmitted primarily by *Aedes aegypti* and secondarily by *Ae*. *albopictus* [5], in predominantly urban human transmission cycles [6]. *Ae*. *aegypti* prefers human blood [7–9]; is more likely to oviposit in water storage containers than natural depressions [10, 11]; and its eggs withstand desiccation for more than four months, on average, in high humidity settings [12]. Common breeding habitats include containers for water storage; domestic and cookware receptacles; flower pots; and discarded objects, including tires and trash [13]. As a result, *Ae*. *aegypti* flourishes in crowded human settlements lacking access to piped water, waste collection, and adequate health and vector control systems. Such areas typify expansive and unplanned urban peripheries in tropical and subtropical latitudes [14]. In those settings, even when access to piped water is prevalent, intermittent and unreliable provision may compel populations to store water in containers, introducing *Aedes* oviposition sites [15]. High population density (and thus easy availability of a blood meal or breeding site) and anthropogenic constraints on urban low-level flight (such as highways, railroads, and buildings), likely limit mean *Ae*. *aegypti* flight range to < 200m in urban settings [10, 16, 17]. Notwithstanding average and maximum flight distances, adult *Ae*. *aegypti* remain in close proximity to the site of their larval/pupal development [18], predominantly concentrating and transmitting DENV indoors [19], such that a substantial share of viral circulation is believed attributable to the socially structured movement of human hosts through areas where they are exposed to DENV-infected mosquitoes [20, 21].

A variety of vector control strategies are available, targeting different stages of the mosquito [22], as well as educational campaigns aimed at promoting behaviors that minimize the proliferation of breeding habitats [23–25]. The World Health Organization (WHO) endorses an integrated approach to vector control [26], including an ecological, biological, social (‘Eco-bio-social’) dengue transmission model emphasizing community participation, as well as identification of local sources of dengue exposure [23, 27]. A primary challenge to interventions, however, is implementation at scale in a manner that can be sustained across periods of epidemic and interepidemic transmission [28, 29]. Insecticide treated bednets are inadequate to rebuff diurnal, endophilic *Aedes* mosquitoes, and large-scale adulticide fogging has been characterized as a cosmetic measure [30]. There is evidence that treatment of curtains and screens with insecticide reduces *Aedes* density [31, 32] with communal spillover effects [33], and that indoor residual [17, 34] and space [35] spraying reduce both vector density and dengue incidence.

Determining the time, place, and combination of control interventions requires proper knowledge of dengue epidemiology, which is influenced not only by the characteristics of the vector, but also by many biological (e.g., serotype), social (e.g., education), behavioral (e.g., storage of water), economic (e.g., income), and environmental (e.g., climate) factors [15, 36–43]. Temporal trends in dengue incidence within an endemic area are commonly characterized by typical inter-annual and seasonal variability. First, a cyclical inter-annual pattern of epidemic and lower-level interepidemic transmission exists, with as much as a tenfold difference in observed cases year-to-year [35, 44]. Second, regardless of the degree of endemicity, within each year dengue incidence exhibits seasonality, with peaks usually observed during warmer and wetter months [37, 45]. The appearance of DENV genetic variants with greater epidemic potential is linked to sustained urban interepidemic transmission [44, 46], increasing the importance of identifying factors contributing to transmission during these intervals.

This cyclical, seasonal, and context-dependent nature of urban dengue transmission present challenges for targeting control, and a better understanding of possible differences in drivers of transmission within and between interepidemic and epidemic years is needed. This study addresses this need, and aims to assess the role of structural, environmental, and human factors in the spatial and temporal distribution of reported dengue cases in Fortaleza, the 5^th^ largest city in Brazil. The analysis considers five consecutive years (2011 to 2015), capturing both epidemic and interepidemic transmission. Observing the ubiquity of reported dengue infections in Fortaleza during epidemic months of peak transmission, we hypothesize that ecological correlates of infection differ within and between years according to the scale of transmission (epidemic vs. interepidemic) and the season (low vs. high precipitation). If factors associated with dengue incidence in Fortaleza during periods of low transmission are different than those that characterize incidence during epidemic peaks, vector control efforts may inadvertently neglect issues that sustain local viremic circulation between epidemics. Since suppression of interepidemic transmission may be a means to forestall or avert epidemics, identifying conditions that are associated with incidence during each stage of the epidemic cycle represents much needed evidence to inform dengue prevention and control.

## Materials and methods

### Study area

**Fig 1:**
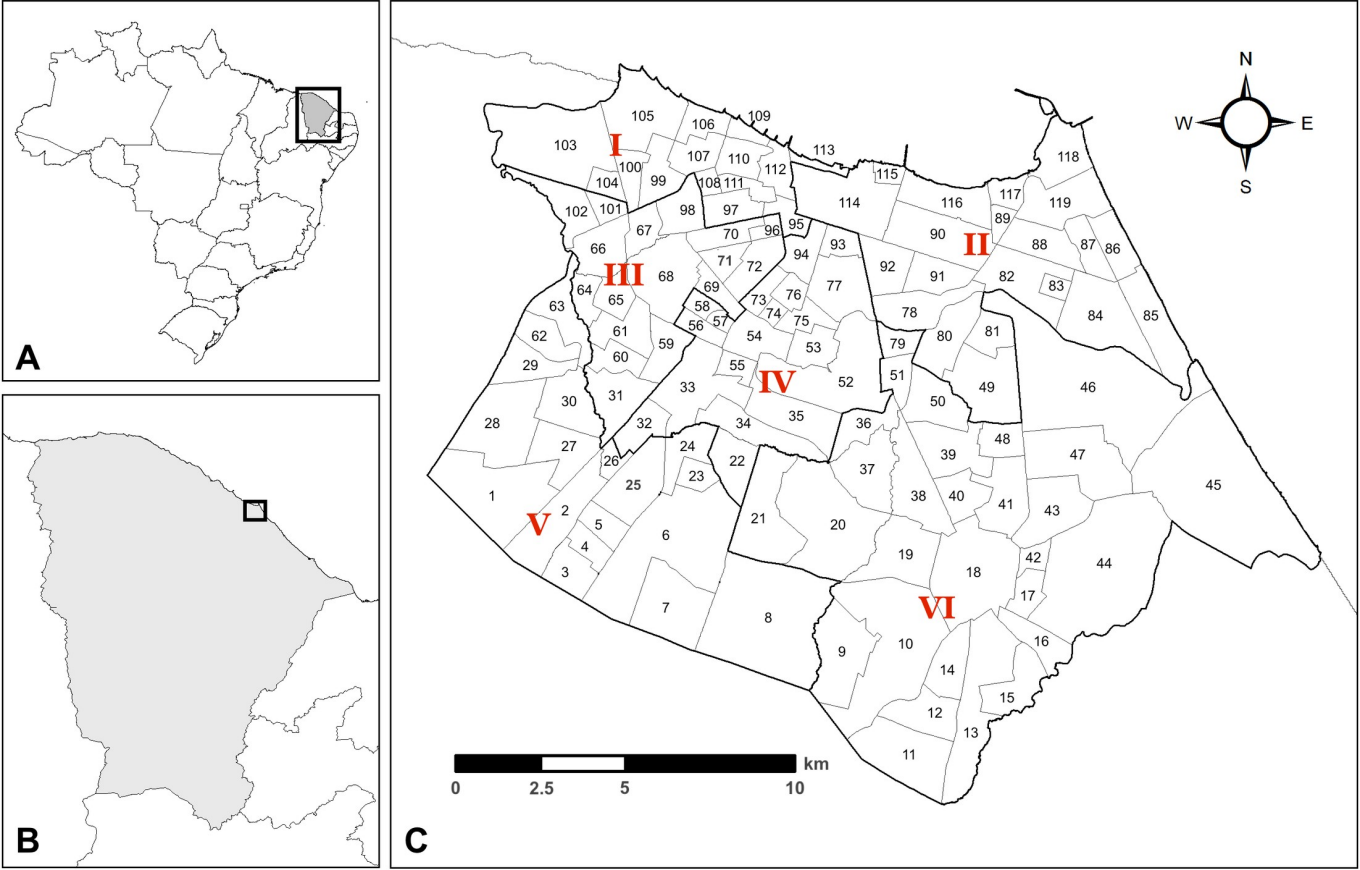
Administrative districts of Fortaleza, Ceará, Brazil. A: Brazil state boundaries; B: Ceará state; C: Fortaleza municipality. Red numerals (I-VI) refer to Fortaleza *regionais* (districts), numbers refer to *bairros* (neighborhoods). A *bairro* name key is included in supplementary material (S1 Table).

Located in the Northeast (NE) region of Brazil, Fortaleza is the capital of Ceará state and the 5^th^ most populous city in Brazil (estimated at 2.6 million inhabitants in 2018) [47]. Fortaleza is divided into six *regionais* (administrative districts) and 119 *bairros* (administrative sub-districts, or neighborhoods) (Fig 1). The United Nations income-based urban inequality index classifies Fortaleza as the 9^th^ most unequal city in the world [48]. The city has the highest homicide rate in Brazil, and the 12^th^ highest homicide rate in the world, with approximately 61 homicides per 100,000 people in 2015 [49].

The Köppen climate classification describes Ceará as equatorial savannah, with dry winters and humid summers [50]. The seasonality of annual precipitation cycles in Ceará is modified by the Atlantic Multidecadal Oscillation (AMO) climate cycle, which causes anomalous wet-dry phases lasting for multiple decades [51].

### Dengue transmission in Fortaleza

After the elimination of *Ae*. *aegypti* from most of the Americas in the 1950s –except for the United States, Suriname, Venezuela, and several Caribbean states [52]–the mosquito was reintroduced in Brazil in the late 1970s, at a time when entomological surveillance activities and vector control teams were, for the most part, non-existent [53]. Dengue reemerged in Brazil during the early 1980s, with epidemics of DENV1 in Northern Brazil and Rio de Janeiro state in 1981 [54]. These outbreaks were followed by a series of epidemics in coastal cities of the Southeast (SE) and NE regions, and nearly 300,000 cases were reported nationally between 1986 and 1993 [55]. Between 2000 and 2010, national incidence varied widely by year, ranging from 74,000 cases in 2004 to more than 1 million in 2010 [56].

Following an absence of over fifty years, cases of DENV1 were first recorded in Fortaleza in 1986. DENV1 was the only serotype identified in the city until 1994, when DENV2 was associated with an epidemic of nearly 30,000 cases. DENV1 and DENV2 predominated until 2002, when DENV3 was first observed in the city. While reported annual incidence between 1994 and 2008 did not exceed 20,000 cases, recent epidemics of DENV2 (2008), DENV1 (2011) and allochthonous DENV4 (2012) all exceeded 34,000 reported cases [57].

### Dengue control in Fortaleza

Within the Fortaleza Municipal Health Secretariat, the Vector Control Program coordinates activities to prevent and respond to dengue epidemics in accordance with national protocols. The municipality employs approximately 1,260 individuals to conduct *Ae*. *aegypti* monitoring and control activities [58]. Epidemiological surveillance and vector control departments carry out three to four sampled entomological surveys annually to estimate property infestation, and visit targeted surveillance sites (called strategic points) every fifteen days. In parallel with epidemiological surveillance, Vector Control Program agents conduct community canvassing operations throughout the year to identify and destroy breeding sites, chemically treat potential breeding sites that cannot be destroyed (e.g. elevated water tanks and cisterns), and implement public information campaigns to promote community-based vector control [59].

### Data sources

This study assembled data from multiple sources. Dengue cases recorded in Fortaleza between 2011 and 2015 were acquired from Brazil’s Notifiable Diseases Information System (*Sistema de Informação de Agravos de Notificação*, SINAN) [60]. By law, suspected dengue cases are reported by clinicians and health professionals to SINAN within 24 hours of initial diagnosis [61]. Suspected dengue cases include all episodes of fever lasting two to seven days which are accompanied by at least two symptoms including nausea, vomiting, positive tourniquet test (i.e. capillary fragility test), petechiae, leukopenia, headache, retro-orbital pain, myalgia, or rash; and exposure to areas with active dengue transmission or presence of *Ae*. *aegypti* during the prior fourteen days [62]. Within 60 days of reporting, suspected cases are coded by the Municipal Health Secretariat as discarded/inconclusive (when not all criteria for a suspect case, as described above, was met), or coded as confirmed dengue according to: (i) laboratory analysis–based on results of IgM serology (ELISA), NS1, viral isolation, RT-PCR, or postmortem immunohistochemistry; or (ii) clinical/epidemiological criteria–when a laboratory test was not performed, but all criteria were met [63]. The MoH recommends that as many cases as possible should be confirmed by laboratory analysis, except in epidemic periods, when about 10% of laboratory confirmation is consired to be sufficient to characterize the epidemiological situation [63]. In this study we considered only confirmed dengue cases.

We used data from years 2011 to 2015 to capture both the cyclical and seasonal patterns of dengue transmission: 2011, 2012, and 2015 were considered epidemic years, and 2013, and 2014 were interepidemic years. Case home addresses were geocoded and then aggregated to the *bairro*-level. Sufficient information to geocode cases to *bairros* was available for 97.5% of confirmed cases in 2011, 77.5% in 2012, 98.1% in 2013, 95.4% in 2014, and 77.7% in 2015. Temporally, cases had the exact date of first symptoms, which allowed aggregation to epidemiological weeks and to months. All data analyzed were anonymized.

We obtained annual population data by *bairro* from the 2010 Demographic Census [64] and estimated *bairro* population growth from 2011–2015 using estimates of population growth for the city of Fortaleza from the Brazilian Institute of Geography and Statistics (IBGE). Using dengue cases and population, we calculated incidence rates per 100,000 people for each *bairro*, and created a binary variable to indicate low and high transmission months in each year: (i) high corresponds to months with more than 9% of annual transmission (or more than one would expect if cases were uniformly distributed over the year), or more than 1,000 total reported cases citywide, and (ii) low corresponds to the remaining months.

We obtained *bairro*-level socio-ecological data from the 2010 Demographic Census [64]. These include: mean number of inhabitants per household; percent of households connected to electricity, piped water, sewage, and regular garbage collection; population density; average income per household in *Reais* (R$); and literacy rates for men and women separately. Two new *bairros* were created after 2010 (both originated from larger *bairros* split into two). In those cases, socio-ecological data for the original *bairro* in 2010 was extended to the new *bairros*.

We estimated the degree of structural deprivation in a *bairro* using two data sources. First, we obtained information for subnormal agglomerations (AS, *aglomerados subnormais*) by census tract from the 2010 Demographic Census. AS is a habitation class characterized by tenuous legal claim and poor structural development. An AS is constituted by, at minimum, 51 inhabited units on illegally occupied land or land obtained within the prior ten years; constructed haphazardly or outside preexisting structural standards; and lacking access to essential public services such as electricity, garbage collection, water grids, and sewage systems [65]. Second, from the Planning Institute of Fortaleza (IPLANFOR, *Instituto de Planejamento de Fortaleza*) [66], we obtained data on precarious settlements (AP, *assentamentos precários*). AP is an exclusively urban classification introduced by the National Housing Secretariat (*Secretaria Nacional de Habitação*) that extends the AS definition to include settlement types that are structurally insecure, neglected, or unplanned, even if legally occupied [67]. The spatial boundaries of AP and AS were merged, then overlaid on the *bairro* boundaries to estimate the intersecting areal proportion for each *bairro*. We refer to this aggregate class as subnormal settlements (SS).

Annual homicide counts by *bairro* were collected from the Mortality Information System (*Sistema de Informações de Mortalidade*, SIM), and used to calculate a homicide rate per 10,000 people. It is established that interpersonal violence can deter and disrupt provision of human services by introducing risk for individuals providing and receiving health-related services [68]. As such, we hypothesized that *bairro*-level homicide rates are associated with spatial heterogeneity in access to health and vector control services, and with higher dengue transmission.

Entomological surveillance data were acquired from *Ae*. *aegypti* infestation surveys, conducted by Brazilian Municipal Health Secretariats at a local level. The *Aedes aegypti* Infestation Rapid Survey (*Levantamento Rápido de Índice para Aedes aegypti*, LIRAa) is a sample survey that randomly selects properties for inspection for immature forms (mosquito larvae and pupae). Municipalities are divided into areal groups of between 8,100–12,000 properties, from which a sample of 450 properties is drawn. LIRAa was introduced in Fortaleza in October 2011 to replace the *Ae*. *aegypti* Infestation Survey (*Levantamento de índice de infestação amostral*, LIA), which covered a much larger sample of properties in the municipality (about 10%), and required considerably more time to complete. LIRAa is repeated three to four times per year, with a national survey conducted every October. The Brazilian Ministry of Health uses three categories of infestation intensity to classify risk: (i) satisfactory, <1% infestation; (ii) alert, 1–3.9%; and (iii) risk of outbreak, >3.9% [62]. We obtained data on all surveys available for LIA in 2011 and for LIRAa from 2011 to 2015. For the purposes of this analysis we used data from the January surveillance of both programs–immediately preceding the early-spring rainy season characteristic of NE Brazil–that is considered the most valuable for forecasting regional epidemic risk. In 2014, the January LIRAa survey was delayed until February, resulting in elevated *bairro* infestation indexes relative to the other years; as a result, we designated 2014 LIRAa results as missing, and used a missingness indicator variable [69]. Under the assumption of representative sampling, we used total houses sampled by *bairro* as a denominator to create a *bairro*-level household infestation variable for each year.

We also used data from surveillance of strategic points (SP)–locations identified by the municipality as higher risk for harboring *Aedes* breeding sites–to assess the epidemiological relevance of *Aedes* infestation at targeted non-residential locations. In Brazil, SP are a central component of national directives for vector control to prevent dengue. Municipal vector control operations agents are tasked with creating a roster of sites likely to be susceptible to mosquito oviposition and inspecting those sites every 15 days. These operations are coordinated locally, such that each municipality is responsible for its own roster of strategic points [62]. Vector control teams inspect SPs in 15-day cycles and code the visit as positive or negative for the presence of *Aedes* larvae or pupae. Between 2011 and 2015, surveillance agents identified 82 types of SPs in Fortaleza; we aggregated those into six categories: construction, industrial fabrication, recyclable processing, scrapyards, sites associated with tire storage–such as tire repair shops and garages, and an “other” category, which included sites associated with vacant lots, public and private facilities (e.g. hospitals and schools), husbandry (e.g. cattle pens, *vacarias*), private residences, outdoor locations (e.g. cemeteries), and general commercial properties.

**Fig 2:**
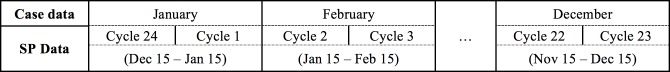
Lag time scheme to relate monthly dengue cases and 15-day inspection cycle of strategic points. Cycles are numbered sequentially, starting with Cycle 1 for the first 15 days of the year.

We created two *bairro*-level variables for each SP category: (i) positivity, defined as the proportion of successfully visited sites that were registered as positive for larvae/pupae; and (ii) proximity, defined as the areal proportion of the *bairro* that falls within a 150-meter buffer area around a positive strategic point. We selected the buffer size using two criteria: (i) expected *Aedes* mosquito flight distance in an urban setting [10, 16, 17]; and (ii) buffer size used by the Brazilian Ministry of Health to define the transmission block area around identified vector oviposition sites [62]. For the positivity variable, *bairros* without an SP visit during an inspection cycle were coded as missing with a missingness indicator variable. The proximity variable was calculated in ArcGIS. To properly associate the presence of mosquito larvae/pupae with monthly dengue virus transmission, SP-related covariates were lagged by a 15-day cycle, as shown in Fig 2.

We acquired rainfall data from the Climate Hazards Infrared Precipitation with Stations (CHIRPS) dataset [70]. CHIRPS has a spatial resolution of 0.05°, thus Fortaleza was covered by 15 pixels. Daily precipitation estimates were aggregated to weeks, and spatially joined to *bairros*. Efforts to associate precipitation volume with dengue incidence must incorporate a time lag to adjust for vector development. While lags of zero [45], one [71, 72], and two months [37, 73] were reported, the mechanistic role of precipitation in dengue transmission can be confounded by water storage behavior [43], and with the potential for heavy rains to wash out exposed oviposition sites [74]. For the purposes of our analysis, precipitation was included as a continuous variable (monthly sum in millimeters), and lagged by two weeks, consistent with the lag used with the SP data. To assess potential effects of rainfall anomalies [75] during the period, we calculated the average monthly rainfall by *bairro* over the five years of study and computed the deviation of each *bairro*-month from its five year average. This variable was included in all models, except those stratified by monthly precipitation volume.

Lastly, daily temperature values (high, low, and daily average) recorded at Fortaleza’s Pinto Martins International Airport were obtained from the Weather Underground data archive [76] and used to generate two measures of temperature variability over the course of a month [77]: monthly average of daily temperature ranges (high minus low daily temperature), and monthly standard deviation of daily temperature averages.

### Analytical approach

We defined the outcome variable as the monthly case incidence rate (dengue cases per 100,000 people) by *bairro*, observed from January 2011 to December 2015, and used a zero-inflated negative binomial longitudinal regression model (hereafter, longitudinal model). The zero-inflated negative binomial model accounts for extra-variation (overdispersion) in the data [78, 79], and was considered as an alternative to the negative binomial hurdle model given the likelihood that seasonally inflated zeros are attributable to both true changes in ecological dynamics that depress transmission, as well as reduced surveillance or misclassification during low seasons and interepidemic years [80–82]. The final model was selected based on comparison of Akaike Information Criterion (AIC) values for alternative distributions, including negative binomial, poisson, and zero-inflated poisson. We chose longitudinal models because our data are longitudinal at the *bairro* level, and used Huber-White standard errors [83]. Climatological and ecological independent variables–selected based on previously identified associations with *Aedes*-borne disease transmission and summarized by *bairro*-month–included: mean household size; percentage of households with access to electricity; percentage of households with access to piped water; percentage of households with access to regular garbage collection; percentage of households connected to sewage network; male literacy rate; female literacy rate; household income; population density; proportion of a *bairro* classified as a subnormal settlement; homicide rate; separate lagged strategic point proximity variables for each of the following types: construction, material fabrication, recyclables, scrapyard, tires, and others; lagged total infestation index for all SP categories combined; categories of infestation (LIA and LIRAa); lagged total precipitation (mm) and deviation from average precipitation (mm); and standard deviation of daily temperature averages (degrees Celsius). The AIC was used to select the final temperature covariate, and likelihood ratio test statistics (chi-square) were used to compare the full model with naïve intercept-only models, indicating that the full model was a better fit (p<0.001) for all analyses.

**Fig 3:**
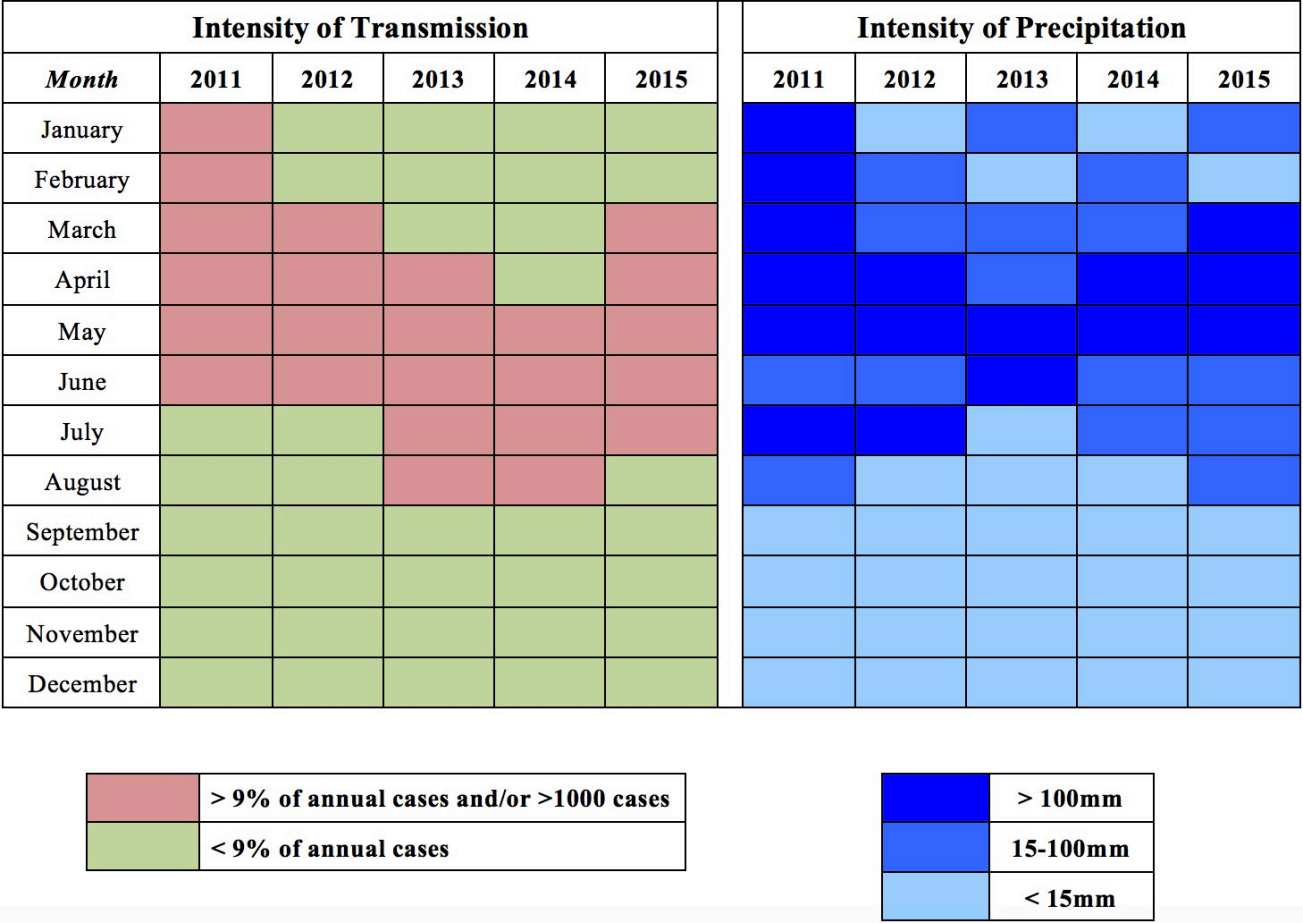
Classification of months according to the intensity of dengue transmission and of precipitation, Fortaleza, 2011–15. Months with high intensity of transmission (more than 9% of total annual dengue cases or more than 1,000 total cases citywide) are represented in pink, and those with low intensity in green. Months with high intensity of rainfall (more than 100 millimeters), medium (15-100mm), and low (less than 15mm) are represented in shades of blue.

Considering the cyclical and seasonal patterns of dengue transmission, we run eight temporally stratified models. Model 1 contains all 7,140 *bairro*-months for which data were collected. Models 2 and 3 distinguish between epidemic (2011, 2012, and 2015) and interepidemic (2013, 2014) years. Models 4 and 5 stratify the analysis by transmission intensity, while Models 6, 7, and 8 are stratified by the intensity of precipitation (Fig 3). We addressed the family-wise error rate in all models using the false discovery rate (FDR) [84]. All data preparation and regression analysis was done in Stata v.14.2 (Stata Corp., College Station, TX, USA).

Lastly, to characterize the spatial and temporal patterns of epidemic intensity between years we used average linkage hierarchical clustering with an Euclidean distance dissimilarity measure, based on eight metrics, standardized as z-scores, reflecting the duration and burden of dengue incidence in a *bairro*. The number of clusters selected for each year was determined with reference to the gap statistic [85], reflecting the difference of within-cluster variability (as the within-cluster sum of squares around cluster means) at each number of *k* clusters in the observed data from its expectation in a null reference distribution computed from repeated sampling using the package factoextra [86]. Values of the gap statistic indicate the strength of clustering at each quantity of clusters considered (i.e. sequential values *k*). Maxima of the gap statistic may be local (in comparison to their immediate neighboring values *k*) or global (in comparison to all possible or considered levels *k*), and a plateau in the statistic indicates the negligible added value of additional partitions between clustering groups [85]. We considered between 3–7 clusters for each year to preserve interpretative value, prioritizing global maxima of the gap statistic (for years 2012, 2013, 2015 –S1 Fig), or local maxima that distinguish between well-separated groups, where further divisions between clusters in the considered range would exclusively partition single-*bairro* clusters (2011). Though the gap statistic first plateaued at three clusters in 2014, we partitioned the middle cluster–with high within-cluster variability along the first principal component of our clustering parameters–to distinguish two clusters, each with low internal variability along that axis (see S2 Fig, clusters II and III). In addition to plots of the first two principal components of clustering parameters (S2 Fig), grouping decisions considered potential informational value to surveillance operations. After grouping, clusters were ordered by the average full-year case rate of clustered units. Clustering analysis and mapping were conducted with R version 3.3.2 [87]. Data used in this study are available in S1 Dataset.

## Results

**Fig 4:**
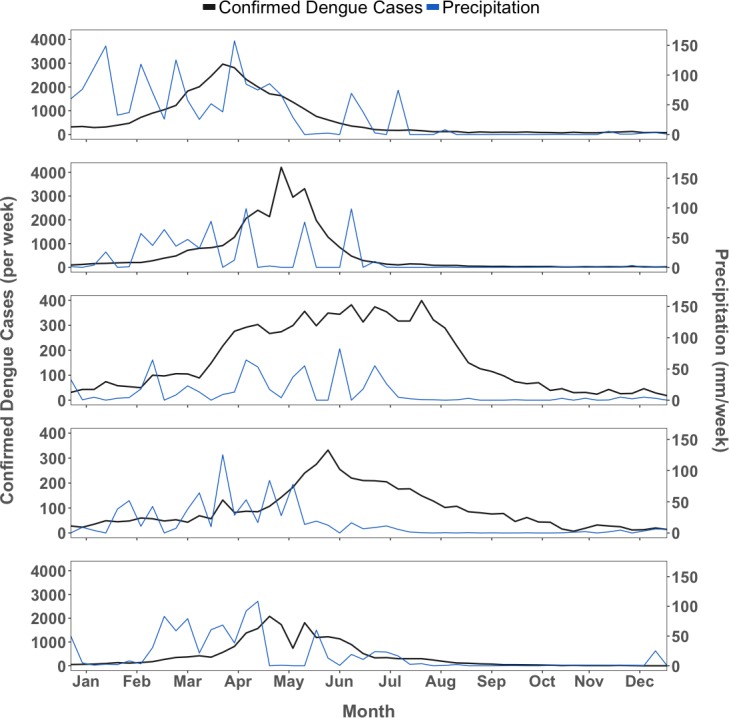
Dengue epidemiological curves and weekly precipitation, Fortaleza, 2011–2015. Average precipitation by week is presented in blue. Line of weekly dengue case count citywide is presented in black. Scale is adjusted for dengue cases (left y-axis) between interepidemic (2013, 2014) and epidemic years (2011, 2012, 2015), and constant for precipitation (right y-axis).

Between 2011 and 2015, 130,430 dengue cases were reported in Fortaleza. In total, 98,339 cases were confirmed by clinical/epidemiological or lab criteria and had sufficient information to be geocoded to *bairros* and included in this analysis. Annual citywide case rates per 100,000 varied from a low of 192 in 2014, to a high of 1,360 in 2011. Monthly rates within a single *bairro* exceeded 3,500 cases per 100,000 people in January 2011, April 2011, and June 2012. In total 26.5% (1,891 of 7,140) of *bairro*-months did not register a case; 75.2% of *bairro*-months with zero cases occurred between August and January, reflecting dengue seasonality. However, the pattern differed between epidemic and interepidemic years, as shown by the percent of annual reported cases that occurred between July and December each year (Table 1 and Fig 4). Incidence during epidemic years was higher, but more concentrated seasonally, while in interepidemic years transmission continued into seasons that were less climatologically hospitable to mosquitoes.

**Table 1:** Number of dengue cases by year, and percent of cases reported between July and December–Fortaleza, 2011–2015.

Year	2011	2012	2013	2014	2015
**Reported Cases**	37,021	34,134	16,381	10,178	32,716
% of total 2011–15	28.4	26.2	12.6	7.8	25.1
% cases in Jul-Dec	9.4	6.0	38.1	39.3	14.4
**Confirmed Cases**	33,642	30,272	8,611	4,935	20,879
% of total 2011–15	34.2	30.8	8.8	5.0	21.2
% cases in Jul-Dec	8.8	5.1	41.8	39.0	13.8

Note: Epidemic years were 2011, 2012, and 2015; interepidemic years were 2013 and 2014.

**Fig 5:**
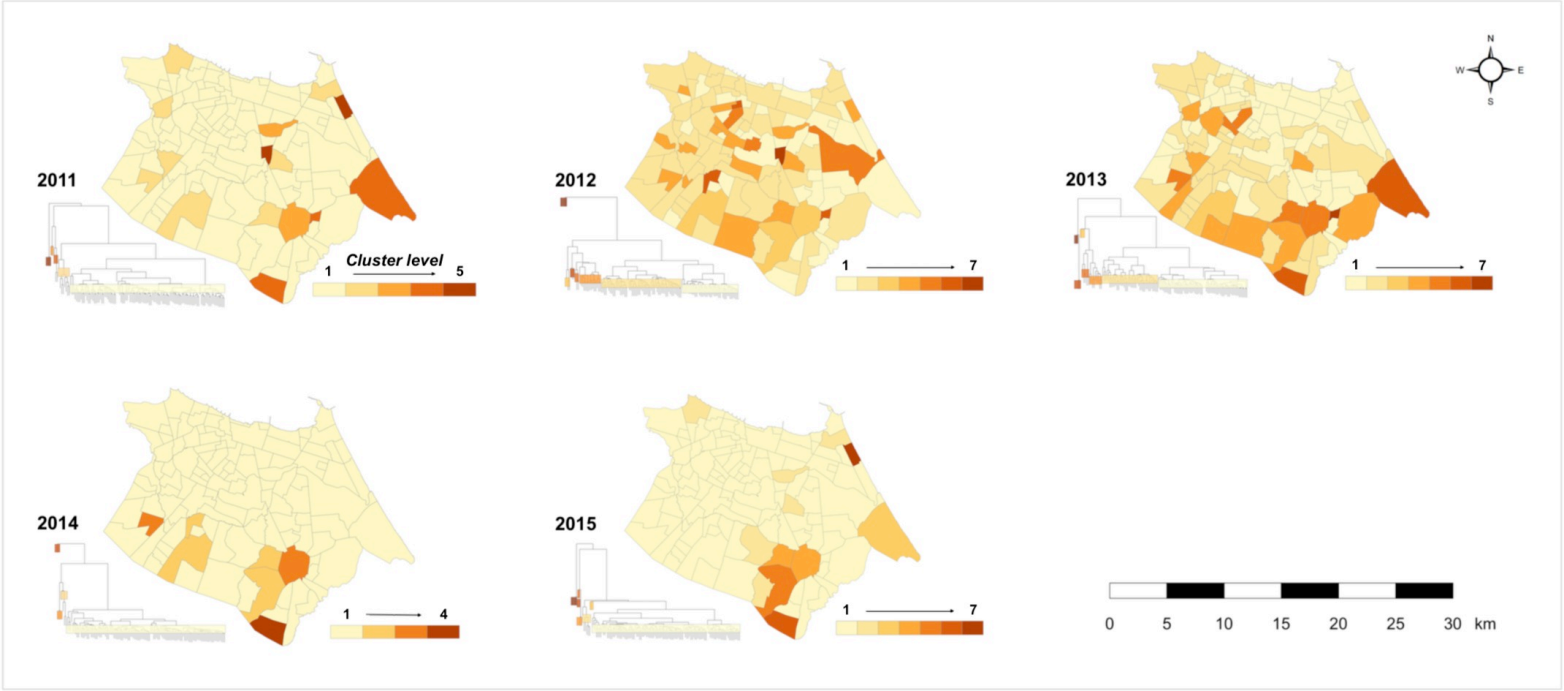
Hierarchical cluster maps of annual dengue transmission intensity in Fortaleza. Averages and ranges of parameter values by year and pattern are presented in S2 Table. Patterns were ordered low-high by annual case rates. Highest level patterns, particularly during lower-transmission years (patterns 6 and 7 in 2013 and 2015; pattern 4 in 2014), classify *bairros* with outlying case rates but shorter transmission intervals. Middle ranked patterns (patterns 2 and 3 in 2011; pattern 3 in 2012; pattern 5 in 2013 and 2015) were characterized by more populous *bairros* with longer intervals with continuous transmission, typically exceeding 40 weeks annually, and higher total case counts.

Hierarchical cluster analysis grouped *bairros* into ascending patterns according to the scale and duration of their dengue burden during annual transmission cycles (Fig 5, S2 Table). Spatially, results suggested that the epidemics of 2011 and 2012 were distinguishable by the increased prevalence of cases in northern, coastal *bairros–*such as the northern coast and northeastern peninsula near Mucuripe–after which incidence concentrated farther from the coastal urban core. Small, outlying clusters (typically composed of only one or two *bairros*) with elevated incidence rates and shorter periods of transmission were present in all years, such that the distance between observations in patterns 1 and 2 was smaller than between other clustering levels, and clustering was strongest during years when a large majority of *bairros* reported minimal dengue transmission (such as 2014 and 2015) (S2 Fig and S3 Table).

In 2011, clustering isolated 15 *bairros* (patterns 2–5), spatially dispersed throughout the city, with substantially elevated or protracted transmission (Fig 5); five of these *bairros* (patterns 4 and 5) exhibited outlying incidence rates (greater than 6,000 per 100,000) over shorter intervals (S2 Table). During the 2011 epidemic, transmission was present or elevated in all *regionais*: two *bairros* in the lowest clustering level experienced more than 3,000 cases per 100,000, over 44 and 48 weeks with confirmed incidence.

The 2012 epidemic and 2013 interepidemic years featured lower levels of transmission overall and fewer outlying *bairros*, decreasing clustering distances between high and low patterns. As a result, clustering distinguished additional patterns at both high and low scales of transmission. Similar to the 2011 epidemic, transmission during the 2012 epidemic was spatially diffuse, with coastal, affluent *bairros* grouped amongst peripheral communities in pattern 2. In contrast, in 2013 northeastern coastal *bairros* were almost exclusively grouped in pattern 1 (Fig 5).

This trend continued in subsequent years, when outlying transmission was either largely isolated (2014) or concentrated (2015) in *bairros* of the southern periphery. Confirmed incidence was negligible in 112 *bairros* of pattern 1 in 2014, yet low level residual dengue circulation is evident in six *bairros* of patterns 2 and 3 (Messejana, Jangurussu, Barroso, Maraponga, Bom Jardim, and Mondubim), where cases were confirmed for 38.5 and 44.5/52 weeks, on average. The scale of annual transmission in these six *bairros* is comparable to pattern 1 *bairros* in 2011, 2012, and 2015, but occurred over much longer intervals (S2 Table). Confirmed dengue incidence in three of these bairros (Messejana, Jangurussu, and Barroso) increased to epidemic levels in 2015 (identifiable in 2015 patterns 4 and 5), inflating case rates for the city as a whole.

Table 2 presents descriptive statistics. Mean household size in Fortaleza was 3.42 persons. Access to infrastructural and municipal services was high (except for sewage), though unequally distributed. Reported literacy exceeded 86% for both men and women in all *bairros*. Minimum reported average annual household income by *bairro* was R$ 800 *reais* (approximately US$450, 2010 exchange rate). Population density exceeded 100 persons per km^2^ in all *bairros* ranging from 148 in Sabiaguaba to more than 34,000 persons per km^2^ in Pirambu. Ten *bairros* included less than 1% SS in their area, and 13 *bairros* had more than 50% SS (highest values of 74%, 87%, and 92% observed in Genibau, Pirambu, and Curio). Homicide rates by *bairro* ranged from zero to 585.6 per 100,000 persons per year; rates for the whole city ranged from a low of 48.33 per 100,000 in 2011 to 76.93 per 100,000 in 2014, exceeding regional averages.

**Table 2:** Descriptive statistics of the *bairro*-level variables used in the analysis.

Variable			Mean	SD	Range
*Reported dengue cases by bairro-month (outcome)*					
Count (number of cases)	2011		23.56	48.52	(0–531)
	2012		21.20	51.11	(0–568)
	2013		6.03	10.29	(0–108)
	2014		3.46	7.68	(0–119)
	2015		14.62	38.17	(0–548)
Rate (cases per 100,000)	2011		127.33	291.55	(0–4,628.3)
	2012		105.43	255.89	(0–3,660.9)
	2013		28.76	48.81	(0–666.9)
	2014		15.87	29.44	(0–355.4)
	2015		71.33	184.12	(0–3,197.3)
*Demographic (exposure)*					
Mean Household size (persons)			3.42	0.19	(2.9–4.0)
Access to electricity (%)			99.65	0.55	(95–100)
Access to piped water (%)			93.02	6.32	(55.8–99.5)
Access to regular garbage collection (%)			98.42	3.33	(78.2–100)
Access to sewage (%)			57.99	33.47	(0.5–99.9)
Literacy (%)	Male		93.61	3.39	(86.4–99.1)
	Female		93.69	2.94	(87.5–98.5)
Household income (R$)			2,532.13	1,970.8	(803.4–10,504.7)
Population density (persons/km^2^)			10,929.1	5,817.8	(148–34,007)
Homicide rate (per 100,000)	2011		48.33	48.25	(0–369.0)
	2012		67.06	60.37	(0–340.2)
	2013		76.91	59.60	(0–262.1)
	2014		76.93	78.11	(0–585.6)
	2015		59.82	51.64	(0–423.1)
Subnormal Settlement (% of *bairro*)			18.28	21.03	(0–91.8)
*Climate (exposure)*					
Precipitation (sum in mm)	2011		138.27	139.19	(0–385.4)
	2012		53.48	63.89	(0–209.6)
	2013		48.67	53.86	(0–171.6)
	2014		64.23	77.50	(0–269.2)
	2015		70.65	82.12	(0–248.3)
Temperature (°C)	Daily average	2011	26.19	0.53	(25.4–27.1)
		2012	26.92	0.51	(26.1–27.9)
		2013	27.38	0.61	(26.3–28.3)
		2014	27.07	0.39	(26.4–27.7)
		2015	27.07	0.60	(26.1–28.2)
	SD of daily averages (by month)	2011	0.86	0.34	(0.51–1.57)
		2012	0.70	0.24	(0.48–1.35)
		2013	0.66	0.22	(0.41–1.11)
		2014	0.64	0.19	(0.35–0.96)
		2015	0.68	0.26	(0.40–1.28)

**Fig 6:**
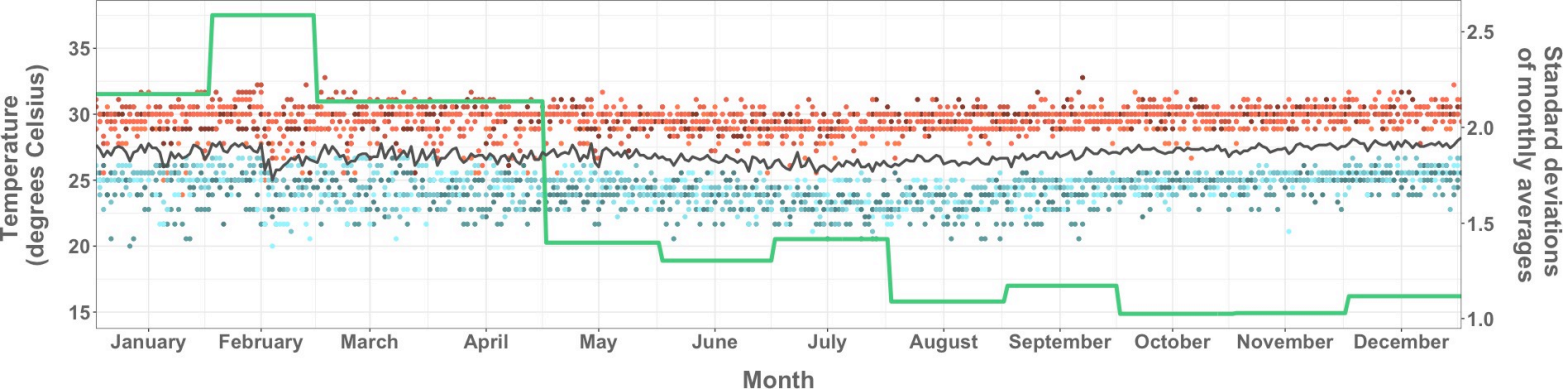
Daily temperature highs and lows, daily average temperature for the 5-year interval, and standard deviation of daily averages by month, Fortaleza, 2011–2015. Values of daily high, low, and average daily temperatures were obtained from each year. High values are represented by dots colored in shades of red, and low values by dots colored in shades of blue. The black line summarizes the daily average for the 5-year period, and the green line shows the standard deviation of daily averages by month.

Temperature in Fortaleza did not vary substantially over the course of our five year study period, but daily temperature averages were marginally higher earlier in the year; the lowest daily temperature average was 22.2 degrees on January 10, 2011 and the highest daily average was 29.4, on March 3, 2013 (Fig 6). In contrast, precipitation differed seasonally, annually, and between *bairros*. At least one *bairro* registered no precipitation during 76% of our study period (197 weeks), and in 37% (96 weeks) no precipitation occurred anywhere in the city. The highest weekly sum precipitation (226mm) was recorded in the second week of March 2015, and precipitation exceeding 83mm was recorded during 10% of all *bairro*-weeks. The largest *bairro* differential in weekly precipitation was 124.2mm, with an average weekly differential of 16.2mm. Average deviation from monthly averages, measured at the *bairro* level, was highest in February 2011, when average *bairro* precipitation exceeded five-year averages for that month by nearly 240mm. This trend continued for the following three months (with positive deviations exceeding 100mm in March, April, and May), amidst the 2011 epidemic. In comparison, citywide averages during spring months of interepidemic years 2013 and 2014 were starkly lower than five-year averages (April 2013 = -137mm, and March 2014 = -101mm).

Entomological surveillance data is summarized in Table 3. On average, *bairro*-level infestation was higher during the LIA (2011) survey than for LIRAa surveys (2012, 2013, and 2015), though the maximum *bairro*-level infestation value (6.45% of visited sites positive for vector immature forms) was recorded in Vila Ellery in January, 2015. The highest average January *bairro* infestation over the course of the four-years (excluding 2014) was in Cambeba, 3.4%.

**Table 3:** Infestation indices, Fortaleza, 2011–2015.

**Parameter**						**Mean**	**SD**	**Range**
**a. Sample-based Entomological Surveys (January cycle)**								
LIA (%)	2011					1.80	1.04	(0–5.29)
LIRAa (%)	2012					0.80	0.76	(0–3.45)
	2013					1.18	0.94	(0–4.62)
	2014 (excluded from analysis)					2.11	1.76	(0–12.5)
	2015					1.42	1.19	(0–6.45)
**b. Strategic Points (targeted surveillance)**								
**Class**	**Year**	**Number of sites**	**Positivity (% infested)**	**Proximity (% areal proportion)**		
			**Mean**	**SD**	**Range**	**Mean**	**SD**	**Range**
Construction	2011	658	10.89	20.26	(0–100)	1.75	4.20	(0–43.6)
	2012	838	6.87	14.60	(0–100)	1.35	3.28	(0–27.3)
	2013	1,115	6.96	14.17	(0–100)	1.99	4.19	(0–31.4)
	2014	1,225	7.79	16.03	(0–100)	1.13	3.06	(0–27.8)
	2015	1,089	9.64	18.82	(0–100)	1.70	4.11	(0–37.0)
	***Average***	**985**	**8.43**	**16.78**	**—**	**1.58**	**3.81**	**—**
Fabrication	2011	128	9.60	20.79	(0–100)	0.48	1.62	(0–16.0)
	2012	138	8.37	20.03	(0–100)	0.45	1.43	(0–16.0)
	2013	162	9.88	20.33	(0–100)	0.56	1.56	(0–16.2)
	2014	155	10.18	22.54	(0–100)	0.38	1.37	(0–16.2)
	2015	143	9.79	21.23	(0–100)	0.38	1.26	(0–9.2)
	***Average***	**145.2**	**9.56**	**20.98**	**—**	**0.45**	**1.46**	**—**
Recyclables	2011	277	8.05	18.46	(0–100)	0.76	2.21	(0–23.3)
	2012	292	4.43	13.64	(0–100)	0.46	1.55	(0–18.4)
	2013	325	4.38	11.16	(0–100)	0.56	1.77	(0–18.4)
	2014	323	5.96	14.75	(0–100)	0.47	1.49	(0–14.5)
	2015	284	5.18	15.41	(0–100)	0.41	1.52	(0–18.4)
	***Average***	**300.2**	**5.60**	**14.68**	**—**	**0.53**	**1.73**	**—**
Scrapyard	2011	333	9.74	21.61	(0–100)	0.86	2.45	(0–37.7)
	2012	352	7.43	18.32	(0–100)	0.67	1.87	(0–18.0)
	2013	397	7.40	16.81	(0–100)	0.78	2.06	(0–23.3)
	2014	406	7.94	18.30	(0–100)	0.51	1.49	(0–13.8)
	2015	364	9.33	21.26	(0–100)	0.62	1.63	(0–10.8)
	***Average***	**370.4**	**8.37**	**19.26**	**—**	**0.69**	**1.93**	**—**
Tires	2011	975	5.62	12.71	(0–100)	1.84	3.66	(0–30.2)
	2012	1,042	3.46	8.41	(0–100)	1.34	2.92	(0–22.4)
	2013	1,200	3.65	9.24	(0–100)	1.44	2.98	(0–19.5)
	2014	1,110	5.87	14.20	(0–100)	0.85	2.14	(0–19.8)
	2015	829	7.61	16.78	(0–100)	0.88	2.03	(0–18.7)
	***Average***	**1,031.2**	**5.24**	**12.27**	**—**	**1.27**	**2.83**	**—**
Others	2011	236	15.29	24.36	(0–100)	1.33	3.22	(0–40.6)
	2012	226	10.73	22.10	(0–100)	0.87	2.49	(0–32.3)
	2013	284	11.29	21.55	(0–100)	1.04	2.88	(0–42.5)
	2014	303	11.22	21.88	(0–100)	0.64	1.79	(0–22.3)
	2015	262	11.84	22.29	(0–100)	0.79	2.24	(0–19.4)
	***Average***	**262.2**	**12.07**	**22.44**	**—**	**0.93**	**2.58**	**—**
Total	2011	2,607	8.38	12.02	(0–100)	6.59	8.48	(0–70.15)
	2012	2,888	5.38	9.36	(0–100)	4.88	6.89	(0–54.77)
	2013	3,483	5.84	9.09	(0–100)	5.99	7.50	(0–42.46)
	2014	3,522	6.78	12.46	(0–100)	3.80	5.55	(0–35.08)
	2015	2,971	8.91	14.47	(0–100)	4.58	5.92	(0–38.78)
	***Average***	**3,094.2**	**7.06**	**11.48**	**—**	**5.17**	**6.87**	**—**

**Fig 7:**
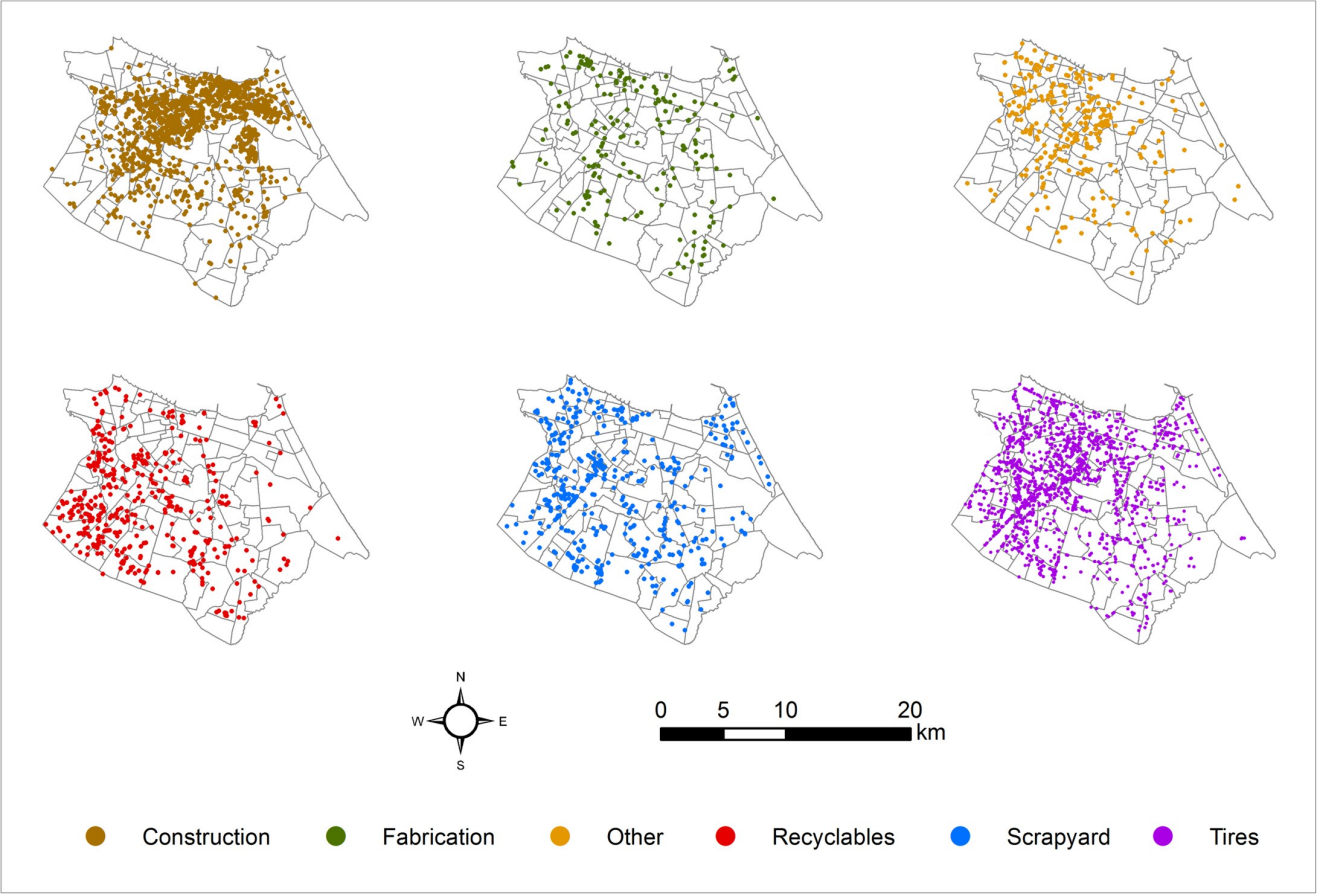
Location of strategic points by type in Fortaleza, 2015.

The spatial distribution of SP types varied across the city (Fig 7 shows their location in 2015), and around 3,000 SP sites were inspected annually, on average, during the study period. The largest number of SP inspected by surveillance agents was 3,522 in 2014, the majority being tire repair shops and garages (Tires category in Table 3). Considering all types of SPs, the highest infestation indices were registered in 2011 (8.38%) and 2015 (8.91%) (Table 3). There were large differences between infestation by site types: on average, sites in the “Others” category registered the highest monthly infestation indices, while those under the tire category had the lowest (Table 3). SP infestation exceeded household infestation (LIRAa/LIA), on average, and was substantially more variable at the *bairro* level. The monthly areal proportion of a *bairro* within 150 meters of a positive SP varied by type of site, reflecting differences in SP prevalence and infestation rates by class. On average, about 5% of *bairro* area was within 150-meter radius of a positive SP citywide, and more than half of this exposure was attributable to sites in the construction and tire categories.

In the analysis of all 7,140 *bairro*-months (Model 1, Table 4), amongst socio-ecological variables only *bairro* homicide rates was a statistically significant correlate of dengue incidence rates: on average, increases in annual household income by R$1,000 were accompanied by an 8% decrease in dengue incidence rates, while an additional ten homicides per 100,000 people was associated with a 6% increase. Over the entire study period, a 1% increase in the areal proportion of a *bairro* within 150 meters of a SP in the tires class was associated with 3% increases in *bairro*-month dengue incidence rates. Indicator variables of epidemic year, peak transmission season, and of precipitation intensity were statistically significant, supporting stratified analyses.

**Table 4:** Longitudinal model considering all *bairro*-months (Model 1).

Parameter		Model 1 (n = 7,140)			
		IRR	95% CI	z	FDR adjusted p-value
**Social & structural factors**	Household size	0.764	[0.419–1.392]	-0.88	0.54
	Electricity	0.956	[0.790–1.158]	-0.46	0.74
	Water	0.995	[0.983–1.007]	-0.87	0.54
	Garbage	0.978	[0.948–1.010]	-1.34	0.33
	Sewage	0.999	[0.995–1.002]	-0.83	0.55
	Literacy (male)	1.100	[0.979–1.236]	1.60	0.26
	Literacy (female)	0.936	[0.828–1.059]	-1.05	0.47
	Income	0.924	[0.865–0.987]	-2.35	0.07
	Pop. Density	0.981	[0.961–1.002]	-1.81	0.19
	Subnormal (%)	1.000	[0.992–1.008]	-0.05	0.97
	Homicides	1.060***	[1.047–1.073]	9.44	<0.01
**Strategic Points**	Construction	1.006	[0.991–1.022]	0.81	0.56
	Fabrication	1.011	[0.986–1.036]	0.83	0.55
	Recyclables	1.013	[0.995–1.031]	1.44	0.30
	Scrapyard	1.019	[1.000–1.038]	1.97	0.14
	Tires	1.032***	[1.018–1.046]	4.49	<0.01
	Other	0.981	[0.961–1.002]	-1.80	0.19
	Total Infestation	0.998	[0.995–1.002]	-0.90	0.53
**LIA/LIRAa**	Alert	1.065	[0.946–1.200]	1.05	0.47
	Risk	1.100	[0.792–1.528]	0.57	0.68
**Climate**	Rainfall (med)	1.240***	[1.145–1.342]	5.31	<0.01
	Rainfall (high)	2.035***	[1.816–2.280]	12.25	<0.01
	Rainfall (deviation)	1.000	[0.999–1.000]	-1.82	0.19
	Temperature	1.424***	[1.238–1.638]	4.95	<0.01
**Scale**	Epidemic Year	2.603***	[2.372–2.856]	20.16	<0.01
	High Month	3.936***	[3.629–4.271]	32.97	<0.01

Results expressed as incidence rate ratios (IRR) associated with a unit change in covariate value. Model adjusted for multiple testing using the false discovery rate (FDR). Significance is represented as:

*** = 0.01.

Tables 5 and 6 show the models stratified by transmission intensity inter-annually (Models 2 and 3) and seasonally (Models 4 and 5). Results indicate that some covariates were strongly associated with dengue incidence rates during periods of low (Models 3 and 5), but not high (Models 2 and 4) transmission. Though income was not a statistically significant correlate of dengue incidence during epidemic years (Model 2), during years of interepidemic transmission a R$1,000 increase in average annual *bairro* household income is associated with reduced dengue incidence by more than 10% (Model 3). Further, while exhibiting a negative, protective effect in all strata, the percentage of properties with regular garbage collection was only statistically significant during interepidemic years (Model 3).

**Table 5:** Longitudinal model stratified by epidemic years and interepidemic years.

Parameter		Annual Transmission Intensity							
		Model 2 (Epidemic) n = 4,284				Model 3 (Interepidemic) n = 2,856			
		IRR	95% CI	z	FDR p-value	IRR	95% CI	z	FDR p-value
**Social & structural factors**	Household size	0.750	[0.382–1.472]	-0.84	0.55	0.807	[0.448–1.451]	-0.72	0.61
	Electricity	0.903	[0.734–1.110]	-0.97	0.50	1.111	[0.897–1.377]	0.96	0.50
	Water	0.985	[0.971–0.999]	-2.15	0.10	1.009	[0.996–1.022]	1.35	0.33
	Garbage	0.991	[0.953–1.032]	-0.43	0.75	0.954**	[0.921–0.988]	-2.65	0.03
	Sewage	1.000	[0.996–1.003]	-0.14	0.92	0.996	[0.992–1.000]	-2.16	0.10
	Literacy (m)	1.066	[0.941–1.208]	1.00	0.49	1.119	[0.972–1.287]	1.56	0.26
	Literacy (f)	0.955	[0.834–1.092]	-0.68	0.63	0.930	[0.800–1.080]	-0.95	0.50
	Income	0.959	[0.883–1.042]	-0.99	0.50	0.893***	[0.844–0.946]	-3.89	<0.01
	Pop. Density	0.978	[0.955–1.002]	-1.79	0.19	0.987	[0.968–1.007]	-1.23	0.39
	Subnormal (%)	1.001	[0.993–1.010]	0.26	0.85	0.997	[0.988–1.005]	-0.81	0.56
	Homicides	1.076***	[1.057–1.094]	8.24	<0.01	1.044***	[1.030–1.057]	6.45	<0.01
**Strategic Points**	Construction	0.995	[0.980–1.011]	-0.59	0.67	1.019	[0.998–1.040]	1.81	0.19
	Fabrication	1.008	[0.983–1.033]	0.61	0.66	1.006	[0.976–1.038]	0.40	0.76
	Recyclables	1.019	[0.996–1.042]	1.58	0.26	1.019	[0.996–1.043]	1.59	0.26
	Scrapyard	1.022	[1.002–1.042]	2.14	0.10	1.028	[1.003–1.053]	2.22	0.09
	Tires	1.033***	[1.016–1.051]	3.73	<0.01	1.017	[0.995–1.040]	1.51	0.28
	Other	0.983	[0.960–1.006]	-1.45	0.30	0.979	[0.959–0.999]	-2.01	0.13
	Total	0.996	[0.991–1.000]	-1.76	0.20	1.001	[0.996–1.006]	0.29	0.83
**LIA/** **LIRAa**	Alert	1.194**	[1.042–1.368]	2.55	0.04	0.944	[0.791–1.128]	-0.63	0.65
	Risk	1.344	[0.978–1.849]	1.82	0.19	1.541	[0.727–3.263]	1.13	0.43
**Climate**	Rainfall (sum)	1.006***	[1.005–1.007]	11.71	<0.01	0.999	[0.999–1.000]	-1.57	0.26
	Rainfall (deviation)	0.994***	[0.993–0.995]	-10.48	<0.01	1.003***	[1.002–1.004]	6.30	<0.01
	Temperature	1.641***	[1.368–1.970]	5.32	<0.01	2.245***	[1.779–2.833]	6.81	<0.01
**Scale**	High Month	3.672***	[3.219–4.189]	19.36	<0.01	2.944***	[2.692–3.219]	23.70	<0.01

Results expressed as incidence rate ratios (IRR) associated with a unit change in covariate value. Models adjusted for multiple testing using the false discovery rate (FDR). Significance is represented as:

** = 0.05

*** = 0.01

**Table 6:** Longitudinal model stratified by high and low transmission months.

Parameter		Monthly Transmission Intensity							
		Model 4 (High, peak) n = 4,284				Model 5 (Low, residual) n = 2,856			
		IRR	95% CI	z	FDR p-value	IRR	95% CI	z	FDR p-value
**Social & structural factors**	Household size	0.847	[0.432–1.660]	-0.48	0.73	0.705	[0.402–1.235]	-1.22	0.39
	Electricity	1.119	[0.943–1.328]	1.29	0.35	0.830	[0.670–1.027]	-1.71	0.22
	Water	0.990	[0.977–1.004]	-1.42	0.31	1.002	[0.991–1.014]	0.42	0.75
	Garbage	0.985	[0.950–1.022]	-0.80	0.56	0.964	[0.934–0.995]	-2.24	0.08
	Sewage	0.997	[0.994–1.001]	-1.43	0.31	0.999	[0.996–1.003]	-0.41	0.76
	Literacy (m)	1.043	[0.898–1.210]	0.55	0.69	1.156**	[1.040–1.284]	2.69	0.03
	Literacy (f)	0.994	[0.845–1.168]	-0.07	0.96	0.891	[0.800–0.992]	-2.09	0.11
	Income	0.915**	[0.860–0.973]	-2.84	0.02	0.908**	[0.849–0.971]	-2.83	0.02
	Pop. Density	0.975	[0.954–0.997]	-2.27	0.08	0.990	[0.970–1.010]	-1.01	0.49
	Subnormal (%)	1.001	[0.992–1.010]	0.14	0.92	0.999	[0.992–1.007]	-0.13	0.92
	Homicides	1.073***	[1.056–1.090]	8.56	<0.01	1.046***	[1.035–1.057]	8.23	<0.01
**Strategic Points**	Construction	1.007	[0.992–1.022]	0.91	0.53	1.009	[0.988–1.031]	0.87	0.54
	Fabrication	1.017	[0.989–1.046]	1.18	0.41	1.009	[0.970–1.049]	0.44	0.74
	Recyclables	1.017	[0.993–1.042]	1.40	0.32	1.016	[0.987–1.047]	1.07	0.46
	Scrapyard	1.015	[0.996–1.035]	1.56	0.26	1.030	[0.996–1.064]	1.74	0.21
	Tires	1.029***	[1.013–1.046]	3.46	<0.01	1.030	[1.004–1.056]	2.27	0.08
	Other	0.983	[0.962–1.004]	-1.59	0.26	1.000	[0.971–1.030]	0.01	0.99
	Total	0.997	[0.993–1.001]	-1.50	0.28	0.996	[0.990–1.002]	-1.39	0.32
**LIA/** **LIRAa**	Alert	1.121	[0.963–1.304]	1.48	0.29	1.062	[0.945–1.194]	1.01	0.49
	Risk	1.198	[0.809–1.773]	0.90	0.53	0.974	[0.768–1.236]	-0.22	0.88
**Climate**	Rainfall (sum)	1.005***	[1.004–1.006]	9.28	<0.01	1.003***	[1.002–1.004]	5.84	<0.01
	Rainfall (deviation)	0.997***	[0.996–0.998]	-7.09	<0.01	0.999	[0.998–1.000]	-1.40	0.32
	Temperature	0.644***	[0.513–0.810]	-3.77	<0.01	1.556***	[1.295–1.870]	4.72	<0.01
**Scale**	Epidemic	3.379***	[2.902–3.934]	15.69	<0.01	1.467***	[1.335–1.613]	7.95	<0.01

Results expressed as incidence rate ratios (IRR) associated with a unit change in covariate value. Models adjusted for multiple testing using the false discovery rate (FDR). Significance is represented as:

** = 0.05

*** = 0.01

With respect to SPs, a 1% increase in areal exposure to tires SP sites was associated with 3.3% increased dengue incidence during epidemic years (Model 2). Conversely, though unit increases in exposure to construction, recycling, scrapyard, and tire sites were all positively correlated with dengue rates during interepidemic years, none were statistically significant when corrected for multiple testing (Model 3). Unlike every other model, the continuous variable for monthly sum precipitation (mm) was not positively associated with incidence over the course of interepidemic years in our sample, reflecting the absence of typical dengue seasonality during those years.

Models 4 and 5 (Table 6) present results stratified by monthly transmission intensity (Fig 3). Though homicides were associated with dengue rates in all models, an additional 10 homicides per 100,000 was associated with a 7.3% higher incidence rate during high transmission months (Model 4), and 7.6% higher rate during epidemic years (Model 2), exceeding increases of 4.6% and 4.4% estimated for periods of lower transmission (Models 3 and 5). Income was a statistically significant protective correlate during both high and low transmission months (Models 4 and 5), and–though not statistically significant when corrected for multiple testing–models estimated stronger associations between regular garbage collection and dengue incidence during late-season months of residual transmission (Model 5). Finally, literacy was a statistically significant *bairro*-level correlate of dengue incidence rates during low months (Model 5), but with divergent effects: male literacy was associated with increased dengue incidence rates, while female literacy was correlated with lower rates. The strongest associations between proximity to infested SPs and dengue incidence related to scrapyards and tire sites within both seasonal strata (Models 4 and 5); while the estimated effects were larger during residual months (Model 5), the corrected associations were not statistically significant. Movement from satisfactory (<1% property infestation) to alert (1–3.9%) levels of larval infestation, as measured by cross-sectional LIRA and LIA surveys conducted in January, was a statistically significant correlate of incidence in *bairros* across epidemic years (Model 2), but not when the sample was isolated to peak months (Model 4). Temperatures were slightly lower and more variable, on average, during epidemic years (Table 2); nevertheless, a negative correlation between variability and incidence during months of seasonal transmission (Model 4) suggests lower temperature variance during peak months of epidemic years relative to interepidemic years.

Models 6, 7, and 8 are stratified by precipitation intensity and presented in Table 7. Consistent with results for low-transmission months (Model 5), literacy and garbage collection were correlates of dengue incidence only during months with minimal rainfall (Model 6). Correlation between dengue incidence and income peaked during “transition” precipitation strata (Model 7)–where R$ 1,000 increases in income were associated with an 11% decrease in dengue incidence rates–but the effect was nearly halved during periods of high precipitation (Model 8). Relative to dry months (Model 6), during wetter months (Models 7 & 8) the magnitude of the association with homicides doubled and a negative association with population density increased in magnitude. Though a relationship between tires SP sites and dengue incidence was observed during low precipitation months (Model 6), associations between dengue incidence and SP proximity were strongest amidst intermediate precipitation (Model 7), when marginal increases in the areal proportion for tire and scrapyard sites were associated with over 4% increased incidence rates. The estimated effect for these SP types declined to 2.9% and 1.1%, respectively, during high precipitation periods (Model 8). While temperature variability was a strong predictor of monthly dengue incidence rates during periods with minimal precipitation (Model 6), it registered a null association during wetter months (Model 8).

**Table 7:** Longitudinal model considering data stratified by precipitation volume (mm).

Parameter		*Bairro* Precipitation Intensity											
		Model 6 (Low, <15mm) n = 3,054				Model 7 (Mid, 15-100mm) n = 1,969				Model 8 (High, >100mm) n = 2,117			
		IRR	95% CI	z	FDR p-val	IRR	95% CI	z	FDR p-val	IRR	95% CI	z	FDR p-val
**Social & structural factors**	Household size	0.680	[0.389–1.191]	-1.35	0.33	0.920	[0.452–1.871]	- 0.23	0.87	0.703	[0.345–1.431]	- 0.97	0.50
	Electricity	0.879	[0.680–1.138]	- 0.98	0.50	0.935	[0.737–1.187]	- 0.55	0.69	1.046	[0.865–1.266]	0.47	0.74
	Water	1.001	[0.988–1.014]	0.10	0.94	0.997	[0.984–1.011]	- 0.38	0.77	0.994	[0.977–1.011]	- 0.72	0.61
	Garbage	0.952**	[0.921–0.984]	- 2.89	0.02	0.989	[0.958–1.020]	- 0.71	0.61	0.981	[0.939–1.024]	- 0.89	0.53
	Sewage	0.998	[0.995–1.001]	- 1.17	0.41	1.000	[0.996–1.004]	- 0.09	0.94	0.998	[0.994–1.002]	- 1.13	0.43
	Literacy (m)	1.126	[1.019–1.244]	2.32	0.07	1.079	[0.946–1.230]	1.13	0.43	1.080	[0.917–1.271]	0.92	0.52
	Literacy (f)	0.908	[0.813–1.015]	- 1.70	0.22	0.964	[0.846–1.100]	- 0.54	0.69	0.951	[0.792–1.143]	- 0.53	0.70
	Income	0.918	[0.847–0.995]	- 2.09	0.11	0.890***	[0.836–0.947]	- 3.68	<0.01	0.938	[0.869–1.012]	- 1.65	0.24
	Pop. Density	1.002	[0.981–1.024]	0.17	0.91	0.976	[0.954–0.998]	- 2.12	0.10	0.970**	[0.947–0.994]	- 2.48	0.05
	Subnormal (%)	0.997	[0.990–1.005]	- 0.70	0.61	1.001	[0.992–1.009]	0.19	0.89	1.000	[0.990–1.010]	- 0.03	0.98
	Homicides	1.036***	[1.025–1.047]	6.61	<0.01	1.072***	[1.056–1.088]	9.26	<0.01	1.073***	[1.054–1.093]	7.53	<0.01
**Strategic Points**	Construction	1.008	[0.984–1.032]	0.61	0.66	1.018	[0.997–1.039]	1.67	0.23	1.006	[0.989–1.023]	0.70	0.61
	Fabrication	1.039	[0.984–1.096]	1.39	0.32	1.007	[0.976–1.040]	0.45	0.74	1.013	[0.983–1.044]	0.87	0.54
	Recyclables	1.025	[0.995–1.056]	1.64	0.23	1.013	[0.990–1.037]	1.10	0.45	1.008	[0.983–1.035]	0.64	0.65
	Scrapyard	1.032	[0.981–1.086]	1.21	0.39	1.041***	[1.017–1.066]	3.36	<0.01	1.011	[0.992–1.031]	1.15	0.43
	Tires	1.040	[1.005–1.076]	2.25	0.08	1.041***	[1.024–1.058]	4.85	<0.01	1.029**	[1.008–1.051]	2.71	0.03
	Other	0.972	[0.945–0.999]	- 2.05	0.12	0.987	[0.971–1.004]	- 1.46	0.30	0.980	[0.952–1.009]	- 1.34	0.33
	Total Infestation	0.999	[0.991–1.006]	- 0.37	0.77	0.993**	[0.988–0.998]	- 2.86	0.02	0.999	[0.994–1.003]	- 0.66	0.63
**LIA/LIRAa**	Alert	1.101	[0.952–1.273]	1.30	0.35	0.971	[0.849–1.111]	- 0.43	0.75	1.114	[0.943–1.318]	1.27	0.37
	Risk	0.914	[0.636–1.314]	- 0.48	0.73	1.131	[0.833–1.535]	0.79	0.57	1.186	[0.768–1.832]	0.77	0.58
**Climate**	Temperature	2.114***	[1.663–2.688]	6.11	<0.01	1.306**	[1.078–1.582]	2.72	0.02	1.162	[0.956–1.412]	1.50	0.28
**Scale**	Epidemic Year	1.194**	[1.056–1.351]	2.82	0.02	3.395***	[2.909–3.962]	15.50	<0.01	4.471***	[3.796–5.266]	17.93	<0.01
	High Month	2.706***	[2.404–3.045]	16.50	<0.01	4.075***	[3.658–4.540]	25.48	<0.01	3.562***	[3.069–4.134]	16.71	<0.01

Results expressed as incidence rate ratios (IRR) associated with a unit change in covariate value. Models adjusted for multiple testing using the false discovery rate (FDR). Significance is represented as:

** = 0.05

*** = 0.01.

## Discussion

This study analyzed five years of dengue incidence at fine spatial and temporal scales. One of the most important findings was the distinct seasonal pattern between interepidemic and epidemic years. Our results indicate that dengue epidemiological curves differed according to the intensity of annual transmission: while the pattern of transmission during epidemic years conforms to widely documented seasonality, during non-epidemic years low-level transmission persisted into climatologically inhospitable conditions. Sustained transmission after June could result from relaxed control activity following a high season with minimal transmission. Such lapses during interepidemic periods are likely to compound entomological and epidemiological challenges for the next annual cycle, and call for sustained vector control activities regardless of transmission intensity. *Bairros* in the southern periphery of the city experienced a larger dengue burden than the coastal city center, particularly during interepidemic years, suggesting that sustained, non-seasonal transmission is linked to conditions in those peripheral areas. Socio-ecological indicators of poverty and deprivation were correlated with higher *bairro*-level dengue incidence rates during seasonal and non-seasonal months–highest during non-epidemic years–but not across epidemics years. The most pronounced associations between *Ae*. *aegypti* surveillance at targeted sites and dengue incidence occurred during transitional precipitation seasons, suggesting that they may be one factor linking stages of residual and epidemic transmission. In contrast to the SP class covariates (which quantify the proportion of a *bairro* spatially proximate to an infested site in bi-monthly intervals), measures of the SP infestation index and entomological cross-sectional surveys (LIA and LIRAa)–ostensibly conducted to identify areas with heightened vulnerability to dengue transmission–did not reliably capture the variability in risk between seasons or *bairros*, corroborating other studies [88, 89]. These results are not surprising, given that the relationship between larval indices and adult densities is diminished by adult flight [90], and variable survival rates of immature forms and productivity by container type [91]. In fact, the association between cross-sectional entomological surveys (LIRAa/LIA) and incidence during epidemic years was dependent upon the inclusion of the precipitation deviation covariate in the model, reinforcing the need for closer scrutiny of those surveys. These findings have direct programmatic implications, identifing places and stages of the epidemic cycle when targeted community interventions can be prioritized by the Fortaleza Municipal Health Secretariat.

While the contribution of non-residential sites (such as the SPs) to vector propagation has been discussed [92], minimal attention is given to their epidemiological relevance. In some cases, non-residential surveillance has been limited to natural sites and niches uncharacteristic of *Ae*. *aegypti* oviposition [93] or a restricted class of non-residential sites, such as schools [94, 95]. Consideration of non-residential structures and spaces that sustain oviposition during dry seasons–such as vacant lots [96], septic tanks [97], and drains [98, 99]–is sparse. Although special surveillance of SPs is mandatory in Brazil, the absence of rigorous examination of the importance of SP infestation for dengue virus transmission limits the capacity of municipal actors to take evidence-based steps to improve their routine control activities. To the best of our knowledge, this is the first study to use fine scale information on SP inspection.

Infestation of SPs was spatially and temporally associated with dengue incidence in Fortaleza, and the magnitude of the associations differed by SP type and according to the scale of transmission and precipitation. Specifically, scrapyards and sites associated with tire collection and storage–such as repair shops and garages–showed associations with dengue incidence during periods of both interepidemic and epidemic transmission. The proportion of a *bairro* proximate to sites known for storing tires was a strong and reliable correlate of dengue incidence within nearly all strata, but the magnitude of the coefficient was largest during transitional precipitation regimes (when rainfall was neither sparse nor extreme) and dry seasons. Further, after adjustment for FDR, scrapyards registered statistically significant correlations with dengue incidence rates during the same intermediate rainfall strata. These results, combined with the new findings regarding the seasonal pattern of dengue transmission in interepidemic years, strongly suggest that enhanced surveillance should be sustained during low transmission periods, in both epidemic and interepidemic years. The Municipal Health Secretariat could characterize scrapyards and tire repair shops as “high risk” strategic points; such a classification could entail more frequent and thorough surveillance proportional to the physical size of the site, and to the number of potential oviposition habitats. An enhanced program would also impose strict and transparent consequences for sustained infestation at these locations (such as revocation of licenses or more effective fine enforcement), and where operators exhibit disregard for vector control protocol during successive visits.

Further, many of the socioeconomic, structural, and environmental factors expected to be associated with dengue transmission showed varied significance according to transmission and precipitation intensity. Among the factors related to access to public services, regular garbage collection was the most consistent correlate of dengue incidence, consistent with previous studies [100, 101]. However, we also show that the link is most pronounced during low transmission and low precipitation periods. Empty lots filled with abandoned trash–as well as discarded materials such as cans, plastic bottles, and debris commonly observed in yards of houses and along sidewalks [15]–could preserve desiccation-resistant eggs, which hatch following intermittent late-season rains. Thus, closer property surveillance and scrutiny of empty lots may be an important component of targeted vector control during interepidemic periods to prevent the escalation to epidemic-scale transmission.

With regard to income, negative [102] and null [100] associations between poverty and dengue incidence have been reported for Fortaleza. In contrast, with the exception of the epidemic year (Model 2) and high precipitation (Model 8) models, when incidence was shown to be more widely dispersed throughout the city, our results indicate a persistent concentration of dengue cases in lower income *bairros*. The association is strongest during periods of intermediate precipitation (Model 7), where a unit increase in *bairro* average household income (equal to one half standard deviation) is associated with 11% decrease in dengue incidence. The associations with income were highly statistically significant during months of high and low transmission (Models 4 & 5), but not in the combined dataset (Model 1), demonstrating the importance of stratifying models by transmission scale. Nonetheless, these results need to be interpreted with caution: health care provided by the private sector is largely underreported in SINAN, despite the fact that notification is mandatory [103]. In Fortaleza, approximately 30% of the population uses private health services, and in more affluent *bairros* this proportion reaches 85%. While the results observed may reflect underreporting of care provided to higher income populations, when stratified annually (Models 2 & 3) correlations with poverty were isolated to non-epidemic years, suggesting that changing dengue transmission dynamics at different stages of the interannual epidemic cycle also underly this association.

Consistent and large correlations between dengue incidence and interpersonal violence, which was also observed for tuberculosis [104], exemplify the difficulty of implementing effective population health measures in expansive cities such as Fortaleza, as well as the importance of involving different governmental sectors to address these challenges. In contrast to many other exposures, homicides were more strongly associated with dengue rates during epidemics and high transmission months. When violence deters actors tasked with providing municipal services it limits access to health services and implementation of responsive vector control [105]. Vector control agents–who are often tasked with canvassing *bairros* and entering properties–may neglect areas that are rife with violence, replicating challenges to effective control of yellow fever that were encountered by Oswaldo Cruz in the early 20^th^ century [106]. In conjunction, wary property owners may refuse entry to vector control and health service officers out of fear for their personal safety [107, 108]. In the context of *Aedes* control, while achieving total coverage is rare, the larger the areas of the city that remain uninspected (and thus untreated), the higher the threat to effective vector control [109].

With regard to *bairro* structural factors, subnormal settlements were not associated with dengue transmission. This may reflect data limitations. Classification of a census tract as AS does not characterize the degree of subnormality, such as the proportion of houses that are subnormal, or the nature of the AS that predominates in a tract. Persistent negative correlations between population density and dengue incidence may reflect the dengue burden in Fortaleza’s peripheral *bairros*–where large expanses without dense development and disconnected from proper urban planning are common (e.g. Prefeito José Walter)–and lower incidence rates in densely populated coastal bairros, whether affluent (e.g. Meireles) or not (e.g. Pirambu).

Though we did not analyze serological data, the Fortaleza Municipal Health Secretariat conducts limited sampling to monitor the introduction of allochthonous serotypes. All four serotypes circulated in Fortaleza between 2011–2015, but DENV1 and DENV4 predominated. DENV1 was reintroduced in 2011 after 10 years, and was the primary serotype in 2011, 2014 and 2015. DENV4 was first introduced in 2012, and was responsible for the majority of cases in 2012 and 2013. In all years, the predominant serotype was present in more than 90% of serological samples, indicating that spatial transmission dynamics may be driven by the degree of population susceptibility throughout Fortaleza’s neighborhoods. As a result of diminished population susceptibility, it is unlikely that DENV1 (which caused an epidemic in 2011) was solely responsible for the estimated disease burden in 2015. The Fortaleza Health Secretariat has found that a significant number of Zika virus (ZIKV) cases were misclassified as dengue in 2015, and that DENV1 and ZIKV co-circulated. Though imported cases of chikungunya (CHIKV) were detected by the surveillance of the City of Fortaleza in 2014, autochthonous cases were not confirmed until December 2015. Therefore, the circulation of ZIKV in 2015 offers one possible explanation for the epidemiological curve observed for that year. The epidemic peak in 2015 was lower than in 2011 and 2012, and incidence in 2015 extended into non-seasonal months at a scale that is more characteristic of interepidemic years (Table 1). It is possible that the peak of 2015 transmission is attributable to seasonal misdiagnosis of ZIKV and CHIKV cases, in which protracted incidence would be characteristic of interepidemic dengue virus transmission in Fortaleza. Alternatively, it is possible that 2015 did have seasonal epidemic dengue transmission, and that protracted non-seasonal transmission is attributable to Zika misdiagnosis.

This study has many strengths. First, data are drawn from multiple years, permitting analysis of interepidemic and epidemic transmission patterns, with and without typical patterns of dengue seasonality. Second, by using temperature and rainfall information, seasonality by year could be properly characterized, accounting for possible weather anomalies. Third, the analysis combined entomological surveys with socio-ecological and climatological data to provide a comprehensive assessment of transmission patterns and correlates. Lastly, the study incorporates routine cross-sectional surveillance surveys of immature forms (considered to be a proxy of the concentration of dengue cases in an urban landscape).

This study has some limitations. First, as any analysis that uses administrative records, data refer to passive surveillance, and do not include asymptomatic infections and/or mild cases that do not trigger search for care [110]. This issue may be qualitatively dependent on the intensity of transmission [56], and communities with access to private healthcare providers may underreport at greater rates than low-income *bairros* [111]. Second, given the circulation of ZIKV in Fortaleza in 2015, the fact that about 20% of cases were lab confirmed in 2015, and the lack of serological tests with high specificity, we expect that some dengue cases reported that year were, in fact, ZIKV infections. Yet, since both diseases are *Aedes*-borne, they are subject to the same socio-ecological factors, and thus their co-circulation should not detract from our conclusions. Third, some variables may not properly capture access and behavior. For example, while we might have expected associations between dengue and access to piped water during dry seasons (as a result of increases in water storage behavior), an ethnographic study in Fortaleza suggested that potable water storage is pervasive [15], such that *bairros*-scale statistics on access to piped water may not be adequate to capture the phenomenon. Similarly, including socio-demographic covariates such as income, access to municipal services, interpersonal violence, and prevalence of subnormal agglomerations at the *bairro* level may not satisfactorily control for socio-economic factors expected to be associated with dengue incidence. As a result, in addition to their role as *Aedes* breeding sites, the prevalence of strategic points–such as scrapyards and tire shops–in a *bairro* may be a proxy for unobserved differences that are relevant to the transmission cycle. Fourth, we only have access to one weather station for temperature data; yet, considering the low variability in temperature observed in Fortaleza, we do not expect this to be a major limitation. Lastly, the entomological surveillance data may only be a partial depiction of the scale of infestation as a result of barriers that vector surveillance agents face when conducting their inspection. Since we are using data from a targeted surveillance program, we assume that some areas may be prioritized because of their known high risk for mosquito breeding.

## Supporting information

S1 Table *Bairro* and regional identification numbers in Fortaleza (ordered by regional). (PDF)

S2 Table Average and range annual values for parameters considered in the hierarchical clustering analysis, by year (2011–2015). (PDF)

S3 Table Standardized principal component loadings and squared loadings of variables used for hierarchical clustering. (PDF)

S1 Fig Gap statistic values calculated from parameters used for hierarchical clustering analysis, by year (2011–2015). Range is bounded at seven clusters. Selected number of clusters identified by vertical dotted line.
(PDF)

S2 Fig Plots of first two principal components of data with selected clustering groups (2011–2015). Outlying single- and multi-*bairro* clusters are labeled.
(PDF)

S1 Dataset Dengue incidence rates and covariates used in the study, by year and *bairro*. (ZIP)
