# Supplementary material for: Epidemiological characteristics and determinants of dengue transmission during epidemic and non-epidemic years in Fortaleza, Brazil: 2011-2015
Source: PLoS Negl Trop Dis. 2018 Dec 3;12(12):e0006990. doi: 10.1371/journal.pntd.0006990 (PMC6292645; doi:10.1371/journal.pntd.0006990)
Supplement: S1 Table — (PDF) [file pntd.0006990.s001.pdf]

S1 Table. *Bairro* and Regional identification numbers in Fortaleza (ordered by regional)

Bairro	ID	*Regional*
Farias Brito	95	1
São Gerardo	97	1
Floresta	99	1
Jardim Iracema	100	1
Vila Velha	103	1
Jardim Guanabara	104	1
Barra do Ceará	105	1
Cristo Redentor	106	1
Álvaro Weyne	107	1
Vila Ellery	108	1
Pirambú	109	1
Carlito Pamplona	110	1
Monte Castelo	111	1
Jacarecanga	112	1
Moura Brasil	113	1
Luciano Cavalcante	49	2
São João do Tauape	78	2
Salinas	80	2
Guararapes	81	2
Cocó	82	2
Cidade 2000	83	2
Manuel Dias Branco	84	2
Praia do Futuro II	85	2
Praia do Futuro I	86	2
De Lourdes	87	2
Papicu	88	2
Varjota	89	2
Aldeota	90	2
Dionísio Torres	91	2
Joaquim Távora	92	2
Centro	114	2
Praia de Iracema	115	2
Meireles	116	2
Mucuripe	117	2
Cais do Porto	118	2
Vicente Pinzon	119	2
Bonsucesso	31	3
Jóquei Clube	59	3
João XXIII	60	3
Henrique Jorge	61	3
Autran Nunes	64	3
Dom Lustosa	65	3
Antônio Bezerra	66	3
Padre Andrade	67	3
Pici	68	3
Bela Vista	69	3
Parquelândia	70	3
Amadeu Furtado	71	3
Rodolfo Teófilo	72	3
Parque Araxá	96	3
Presidente Kennedy	98	3
Olavo Oliveira	101	3
Quintino Cunha	102	3
Dendê	22	4
Vila Pery	32	4
Parangaba	33	4
Itaperi	34	4
Serrinha	35	4
Aeroporto	52	4
Vila União	53	4
Montese	54	4

Bairro	ID	*Regional*
Itaoca	55	4
Demócrito Rocha	56	4
Couto Fernandes	57	4
Pan Americano	58	4
Damas	73	4
Bom Futuro	74	4
Parreão	75	4
Jardim América	76	4
Fátima	77	4
José Bonifácio	93	4
Benfica	94	4
Siqueira	1	5
Canindezinho	2	5
Parque Presidente Vargas	3	5
Parque Santa Rosa	4	5
Conjunto Esperança	5	5
Mondubim	6	5
Planalto Ayrton Senna	7	5
Prefeito José Walter	8	5
Jardim Cearense	23	5
Maraponga	24	5
Manoel Sátiro	25	5
Parque São José	26	5
Bom Jardim	27	5
Granja Lisboa	28	5
Conjunto Ceará II	29	5
Granja Portugal	30	5
Conjunto Ceará I	62	5
Genibaú	63	5
Conjunto Palmeiras	9	6
Jangurussu	10	6
Pedras	11	6
Ancuri	12	6
Paupina	13	6
Parque Santa Maria	14	6
São Bento	15	6
Coaçu	16	6
Guajiru	17	6
Messejana	18	6
Barroso	19	6
Passaré	20	6
Parque dois Irmãos	21	6
Dias Macedo	36	6
Boa Vista	37	6
Cajazeiras	38	6
Cidade dos Funcionarios	39	6
Parque Iracema	40	6
Cambeba	41	6
Curió	42	6
José de Alencar	43	6
Lagoa Redona	44	6
Sabiaguaba	45	6
Edson Queiroz	46	6
Sapiranga Coité	47	6
Parque Manibura	48	6
Jardim das Oliveiras	50	6
Aerolândia	51	6
Alto da Balança	79	6
