# Supplementary material for: Epidemiological characteristics and determinants of dengue transmission during epidemic and non-epidemic years in Fortaleza, Brazil: 2011-2015
Source: PLoS Negl Trop Dis. 2018 Dec 3;12(12):e0006990. doi: 10.1371/journal.pntd.0006990 (PMC6292645; doi:10.1371/journal.pntd.0006990)
Supplement: S2 Table — (PDF) [file pntd.0006990.s002.pdf]

S2 Table. Average and range values of parameters considered in the hierarchical clustering analysis, by year (2011-2015)

**Parameter**			**Total weeks**	**Longest run (weeks)**	**Longest run (count)**	**Max week (count)**	**Full year (count)**	**Longest run (rate)**	**Max week (rate)**	**Full year (rate)**
Year	Pattern	Number of *bairros*	Mean (Minimum-Maximum)							
2011	1	104	31.1 (0-49)	19.7 (0 – 37)	196.5 (0-615)	23.7 (0-76)	216.8 (0-632)	1027.1 (0-3408.9)	133.0 (0-409.0)	1159.8 (0-3588.3)
	2	8	47.4 (39-52)	41.5 (29-52)	747.6 (485-913)	78.4 (35-102)	760.1 (485-948)	1959.4 (663.1-3122.2)	203.6 (47.9-338.4)	1991.3 (663.1-3144.9)
	3	2	46 (41-51)	39 (38-40)	1344 (1045-1643)	139 (130-148)	1360 (1046-1674)	3825.7 (3749.1-3902.2)	419.9 (308.8-531.0)	3864.3 (3752.7-3975.9)
	4	3	26.7 (20-38)	15.7 (8-28)	189 (89-363)	27.33 (18-44)	206 (103-376)	5551.4 (4707.0-6568.3)	944.8 (570.5-1328.4)	6326.1 (4875.5-7601.5)
	5	2	39.5 (39-40)	29.5 (29-30)	822.5 (615-1030)	98 (53-143)	840 (631-1049)	9081.1 (8977.6-9184.6)	1019.0 (791.5-1246.4)	9283.4 (9143.2-9423.5)
2012	1	39	13.5 (0-25)	7.9 (0-18)	31.7 (0-100)	7.7 (0-19)	39.4 (0-109)	370.9 (0-1943.0)	98.3 (0-407.4)	482.0 (0-2381.7)
	2	55	30.3 (20-42)	21.8 (11-33)	250.8 (90-567)	38.8 (11-85)	264.1 (100-582)	1043.2 (207.3-1999.8)	167.9 (20.7-372.3)	1106.9 (213.0-2056.8)
	3	3	46.3 (43-49)	41.3 (39-44)	940.7 (828-1143)	153.3 (141-161)	948.3 (838-1148)	1695.0 (1474.2-2002.1)	285.5 (203.8-378.8)	1709.9 (1480.7-2020.9)
	4	15	37.4 (26-47)	27.3 (19-36)	513 (183-895)	77.8 (30-116)	528.9 (194-910)	2466.0 (1651.7-3180.6)	384.3 (197.4-500.7)	2548.9 (1738.0-3233.9)
	5	3	36.3 (34-40)	23.7 (17-29)	525.3 (511-553)	134.3 (111-169)	546 (533-564)	2785.9 (2261.0-3259.1)	695.1 (631.2-746.3)	2894.2 (2389.0-3399.5)
	6	3	31.7 (25-35)	17 (11-20)	329.3 (241-450)	72 (51-99)	350.7 (259.0-476.0)	3893.5 (3519.8-4346.1)	858.4 (655.0-963.9)	4150.4 (3782.7-4597.3)
	7	1	37 (37)	33 (33)	734 (734)	133 (133)	739 (739)	6337.4 (6337.4)	1148.3 (1148.3)	6380.6 (6380.6)
2013	1	62	14.0 (0-28)	4.7 (0-16)	9.4 (0-47)	3 (0-8)	22.6 (0-72)	52.2 (0-174.5)	21.4 (0-66.4)	135.2 (0-399.1)
	2	39	33.2 (20-43)	16.9 (8-31)	61.3 (26-142)	8.1 (4-13)	92.3 (42-178)	325.8 (74.3-844.3)	46.4 (10.6-101.4)	484.7 (146.0-1031.4)
	3	1	46 (46)	42 (42)	383 (383)	33 (33)	391 (391)	484.0 (484.0)	41.7 (41.7)	494.1 (494.1)
	4	9	38.6 (30-44)	29 (20-40)	175.8 (140-225)	17.6 (14-23)	193.7 (150-245)	472.3 (326.6-613.5)	47.6 (26.7-63.2)	523.0 (349.9-687.8)
	5	5	42.2 (39-47)	34.4 (32-38)	249.4 (174-338)	17.6 (14-22)	262.4 (180-362)	860.1 (666.2-998.0)	64.1 (39.2-90.5)	903.1 (689.2-1032.4)
	6	2	14 (12-16)	5.5 (5-6)	10 (7-13)	4 (3-5)	23 (18-28)	545.8 (501.4-590.1)	220.9 (214.9-227.0)	1280.2 (1271.0-1289.4)
	7	1	19 (19)	13 (13)	87 (87)	21 (21)	119 (119)	1094.8 (1094.8)	264.3 (264.3)	1497.4 (1497.4)
2014	1	112	17.2 (0-37)	6.6 (0-30)	15.9 (0-74)	3.7 (0-11)	31.2 (0-103)	73.6 (0-324.5)	22.4 (0-90.1)	157.4 (0-704.6)
	2	4	38.5 (29-45)	25.7 (17-31)	168.2 (65-271)	18.2 (11-32)	192.7 (82-288)	442.7 (339.8-610.3)	54.9 (28.3-103.3)	525.3 (361.1-769.9)
	3	2	44.5 (43-46)	34.5 (29-40)	311 (282-340)	38.5 (35-42)	326.0 (305-347)	751.8 (645.0-858.6)	92.2 (88.4-96.1)	786.9 (697.6-876.2)
	4	1	11 (11)	5 (5)	7 (7)	5 (5)	19 (19)	497.5 (497.5)	355.4 (355.4)	1350.4 (1350.4)

Parameter			Total weeks	Longest run (weeks)	Longest run (count)	Max week (count)	Full year (count)	Longest run (rate)	Max week (rate)	Full year (rate)
Year	Pattern	Number of *bairros*	Mean (Minimum-Maximum)							
2015	1	107	24.5 (2-41)	16.1 (1-33)	105.6 (1-355)	16.1 (1-47)	119.4 (2-363)	541.7 (8.5-2166.0)	87.8 (8.5-286.2)	635.6 (17.1-2320.7)
	2	6	37.5 (35-40)	30.7 (22-37)	507.2 (348-853)	67.3 (50-95)	522.5 (358-856)	1183.1 (674.3-2205.7)	155.4 (91.5-201.7)	1226.4 (689.9-2213.4)
	3	1	20 (20)	10 (10)	39 (39)	11 (11)	53 (53)	1743.4 (1743.4)	491.7 (491.7)	2369.2 (2369.2)
	4	2	41.5 (39-44)	36 (34-38)	1164.5 (1018-1311)	130.5 (125-136)	1174.5 (1028-1321)	3101.9 (2976.0-3227.7)	352.5 (308.7-396.3)	3129.1 (2998.7-3259.5)
	5	1	44 (44)	41 (41)	1941 (1941)	211 (211)	1945 (1945)	3638.9 (3638.9)	395.6 (395.6)	3646.4 (3646.4)
	6	1	23 (23)	12 (12)	72 (72)	14 (14)	96 (96)	5077.6 (5077.6)	987.3 (987.3)	6770.1 (6770.1)
	7	1	33 (33)	25 (25)	508 (508)	75 (75)	522 (522)	7250.9 (7250.9)	1070.5 (1070.5)	7450.8 (7450.8)

Observations (i.e., *bairros* with values of the clustering parameters) are grouped into clusters according to their Euclidean L2 distance (i.e., dissimilarity) from other observations in the clustering space; these distances correspond with heights in the dendrograms generated from the data and clustering model (Fig 5 in the paper). Observations were initially treated as unique clusters and linked sequentially according to their pair-wise distance. Observations were combined into clustering groups until all observations form a single cluster (the top of the dendrogram). As individual observations form groups, determination of dissimilarity between observation groups was achieved via a selected linkage function; average linkage clustering, used in this analysis, quantifies distance between groups of observations (i.e., formed clusters) as the average pairwise Euclidean distances between all observations in those clusters [1].

The number of clusters selected for each year in Fig 5 was determined with reference to values of the gap statistic [2] for each level of *k* clusters between k=3 and k=7, where the gap values reflect the difference (gap) between a measure of within-cluster dispersion (at each number *k*) W_k in the observed data and the expectation from a null reference distribution (E_n^*) generated from simulations [2]:

$$\mathrm{Gap}_n(k) = E_n^*\{\log(W_k)\} - \log(W_k)$$

$$W_k = \sum_{r=1}^{k} \frac{1}{2n_r} D_r$$

where n_r equals the number of observations in cluster r, and D_r equals the sum of internal pairwise distances. The optimal number of clusters *k* is suggested where Gap(*k*) is greater than the Gap(*k*+1) minus approximately one standard deviation of the replicates $E_n^*\{\log(W_{k+1})\}$:

$$\textit{choose minimum } k \textit{ such that } \mathrm{Gap}(k) \geq \mathrm{Gap}(k+1) - s_{k+1} \text{ [2]}$$

Numbers *k* corresponding with local or global maximums of Gap(*k*) indicate superior partitions of clustering groups, and a plateau in the gap statistic plot represents a level *k* where the creation of additional clustering groups necessitates separating observations from strongly grouped clusters [2].

The analysis was conducted for each year independently, considering between three to seven cluster levels, or patterns. For each year, 150 comparison distributions were simulated. Seven clusters were selected for 2012, 2013, and 2015 based on the global maximum of the gap statistic within this range. A local maximum of five clusters was selected for 2011; this clustering was preferred to the global maximum Gap(*k*=7), which would partition single-*bairro* clusters from patterns III and IV (S2 Fig). Despite the global maximum gap statistic of three clusters in 2014 (within the considered range 3-7 clusters), four clusters were selected to distinguish between multi-*bairro* clusters in patterns II and III (S2 Fig). Estimation and visualization of the gap statistic and plotting with first two principal components (S2 Fig) was completed using the R package ‘factoextra’ [3].

References

1. James G, Witten, D., Hastie, T., Tibshirani, R. An introduction to statistical learning. New York: Springer; 2013.
2. Tibshirani R, Walther, G., Hastie, T. Estimating the number of clusters in a data set via the gap statistic. Journal of the Royal Statistical Society: Series B (Statistical Methodology). 2001;63(2):411-23.
3. Kassambara A, Mundt, F. factoextra. 1.0.5 ed2017. p. R package.
