# Supplementary material for: Epidemiological characteristics and determinants of dengue transmission during epidemic and non-epidemic years in Fortaleza, Brazil: 2011-2015
Source: PLoS Negl Trop Dis. 2018 Dec 3;12(12):e0006990. doi: 10.1371/journal.pntd.0006990 (PMC6292645; doi:10.1371/journal.pntd.0006990)
Supplement: S3 Table — (PDF) [file pntd.0006990.s003.pdf]

## S3 Table. Standardized principal component loadings and squared loadings of variables used for hierarchical clustering

| Year | Component | Loadings (normalized) | | | | | | | |
|---|---|---|---|---|---|---|---|---|---|
| | | **Total weeks 3** | **Longest run (weeks) 1** | **Longest run (count) 2** | **Max week (count) 4** | **Full year (count) 5** | **Longest run (rate) 6** | **Max week (rate) 8** | **Full year (rate) 7** |
| 2011 | 1 | 0.74 | 0.77 | 0.92 | 0.91 | 0.92 | 0.81 | 0.66 | 0.78 |
| | 2 | -0.47 | -0.49 | -0.27 | -0.19 | -0.28 | 0.57 | 0.72 | 0.61 |
| 2012 | 1 | 0.85 | 0.83 | 0.92 | 0.92 | 0.93 | 0.86 | 0.75 | 0.84 |
| | 2 | -0.36 | -0.43 | -0.29 | -0.13 | -0.29 | 0.48 | 0.63 | 0.52 |
| 2013 | 1 | 0.81 | 0.91 | 0.91 | 0.93 | 0.93 | 0.89 | 0.48 | 0.79 |
| | 2 | -0.30 | -0.29 | -0.29 | -0.16 | -0.31 | 0.38 | 0.85 | 0.59 |
| 2014 | 1 | 0.80 | 0.90 | 0.92 | 0.92 | 0.95 | 0.93 | 0.44 | 0.78 |
| | 2 | -0.28 | -0.27 | -0.24 | -0.15 | -0.26 | 0.25 | 0.87 | 0.58 |
| 2015 | 1 | 0.72 | 0.78 | 0.90 | 0.91 | 0.90 | 0.84 | 0.70 | 0.79 |
| | 2 | -0.42 | -0.44 | -0.29 | -0.27 | -0.29 | 0.53 | 0.70 | 0.61 |

| Year | Component | Squared Loadings (normalized) | | | | | | | |
|---|---|---|---|---|---|---|---|---|---|
| | | **Total weeks** | **Longest run (weeks)** | **Longest run (count)** | **Max week (count)** | **Full year (count)** | **Longest run (rate)** | **Max week (rate)** | **Full year (rate)** |
| 2011 | 1 | 0.54 | 0.60 | 0.85 | 0.83 | 0.85 | 0.65 | 0.44 | 0.60 |
| | 2 | 0.23 | 0.24 | 0.07 | 0.04 | 0.08 | 0.32 | 0.52 | 0.37 |
| 2012 | 1 | 0.72 | 0.70 | 0.86 | 0.86 | 0.86 | 0.75 | 0.57 | 0.71 |
| | 2 | 0.13 | 0.19 | 0.09 | 0.02 | 0.08 | 0.23 | 0.40 | 0.27 |
| 2013 | 1 | 0.66 | 0.84 | 0.83 | 0.87 | 0.87 | 0.79 | 0.23 | 0.62 |
| | 2 | 0.09 | 0.08 | 0.09 | 0.02 | 0.09 | 0.14 | 0.72 | 0.35 |
| 2014 | 1 | 0.64 | 0.81 | 0.85 | 0.84 | 0.90 | 0.86 | 0.20 | 0.61 |
| | 2 | 0.08 | 0.07 | 0.06 | 0.02 | 0.07 | 0.06 | 0.76 | 0.34 |
| 2015 | 1 | 0.52 | 0.61 | 0.81 | 0.83 | 0.81 | 0.71 | 0.49 | 0.62 |
| | 2 | 0.18 | 0.19 | 0.08 | 0.07 | 0.08 | 0.28 | 0.49 | 0.37 |

Of the eight variables incorporated in this analysis, two represent duration (weeks), three represent scale (counts), and three represent intensity (rates). Parameter values were calculated for each year independently. For each year, every loading in the first principal component has the same sign, indicating that the first component reflects a correlated characteristic of each parameter, or 'overall burden' [1]. In contrast, the different signs on rate variables in loadings of the second principal component indicate that the second principal component distinguishes the intensity of transmission at the *bairro* scale, predominantly in terms of the parameter "max week" (the highest weekly incidence rate observed in each *bairro* over the course of the year). This pattern was strongest during interepidemic years (0.72 and 0.76 correlation between PC2 and "Max week (rate)" in 2013 and 2014, compared to 0.40 in 2012), reflecting the occurrence of high-intensity weeks during those years (in lower population neighborhoods such as Sabiaguaba and Pedras), notwithstanding low counts over the course of the entire year.

Though duration variables were correlated with PC3, the first two PCs account for more than 90% of variance in every annual subset, indicating that they adequately approximate an *n*-dimensional representation [1]. The only exception to this pattern is 2014, when PC3 accounts for 24.8% of the variance in the primary duration variable "Total weeks" with dengue incidence. This difference – where the duration variable "Total weeks" exhibited lower correlation with count and rate parameters – reflects the anomalous pattern of low-scale, protracted transmission observed during the 2014 interepidemic year in Fortaleza.

**Reference:**

1. Jolliffe IT, Cadima J. Principal component analysis: a review and recent developments. Philosophical Transactions of the Royal Society A. 2016;374(2065).
