# Supplementary material for: Epidemiological characteristics and determinants of dengue transmission during epidemic and non-epidemic years in Fortaleza, Brazil: 2011-2015
Source: PLoS Negl Trop Dis. 2018 Dec 3;12(12):e0006990. doi: 10.1371/journal.pntd.0006990 (PMC6292645; doi:10.1371/journal.pntd.0006990)

S1 Figure. Gap statistic values calculated from parameters used for hierarchical clustering analysis, by year (2011-2015). Range is bounded at seven clusters. Selected number of clusters identified by vertical dotted line.

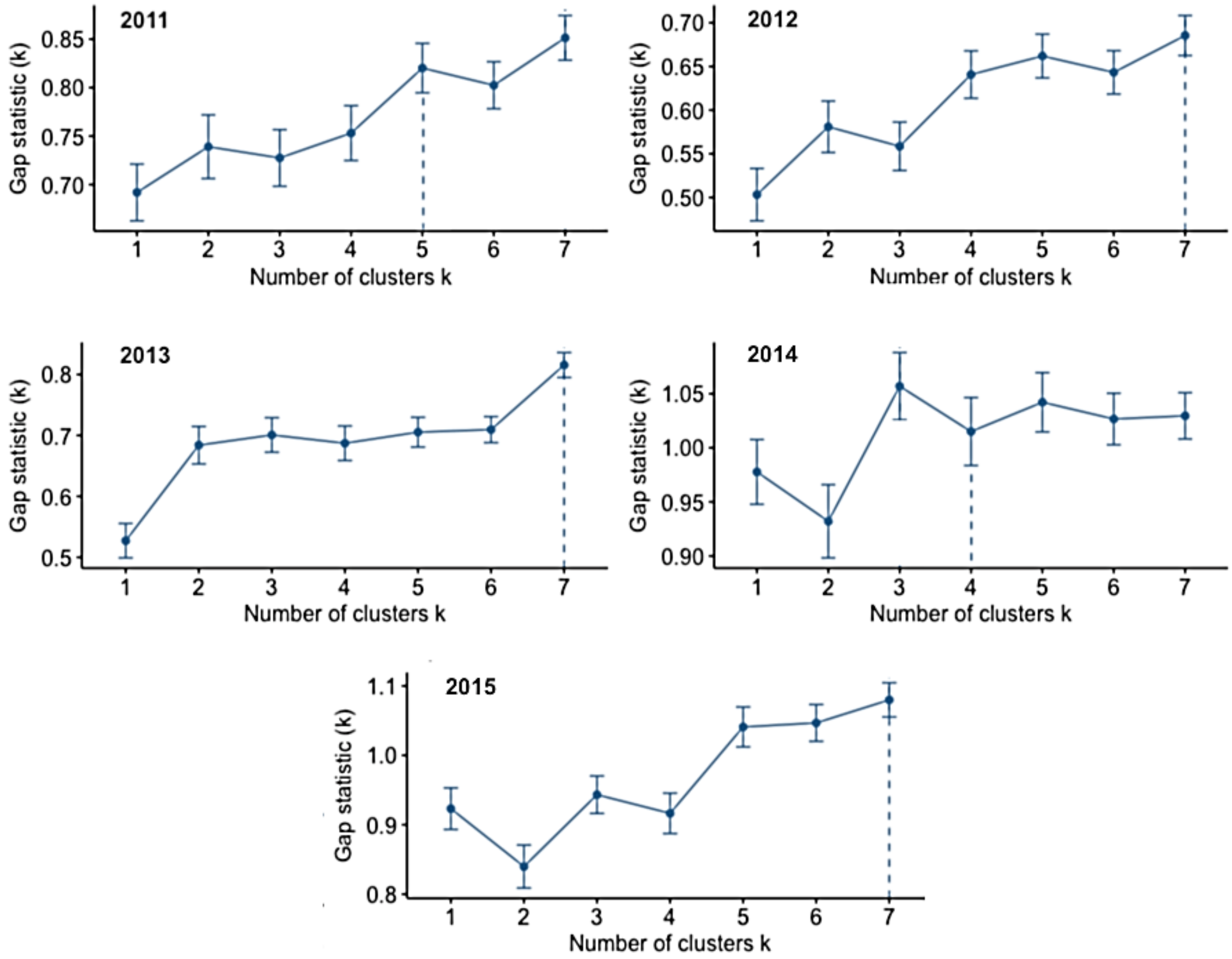

Supplement: S1 Fig — Range is bounded at seven clusters. Selected number of clusters identified by vertical dotted line. (PDF) [file pntd.0006990.s004.pdf]
