# Supplementary material for: Epidemiological characteristics and determinants of dengue transmission during epidemic and non-epidemic years in Fortaleza, Brazil: 2011-2015
Source: PLoS Negl Trop Dis. 2018 Dec 3;12(12):e0006990. doi: 10.1371/journal.pntd.0006990 (PMC6292645; doi:10.1371/journal.pntd.0006990)
Supplement: S2 Fig — Outlying single- and multi-bairro clusters are labeled. (PDF) [file pntd.0006990.s005.pdf]

S2 Figure. Plots of first two principal components of data with selected clustering groups (2011-2015). Outlying single- and multi-*bairro* clusters are labeled.

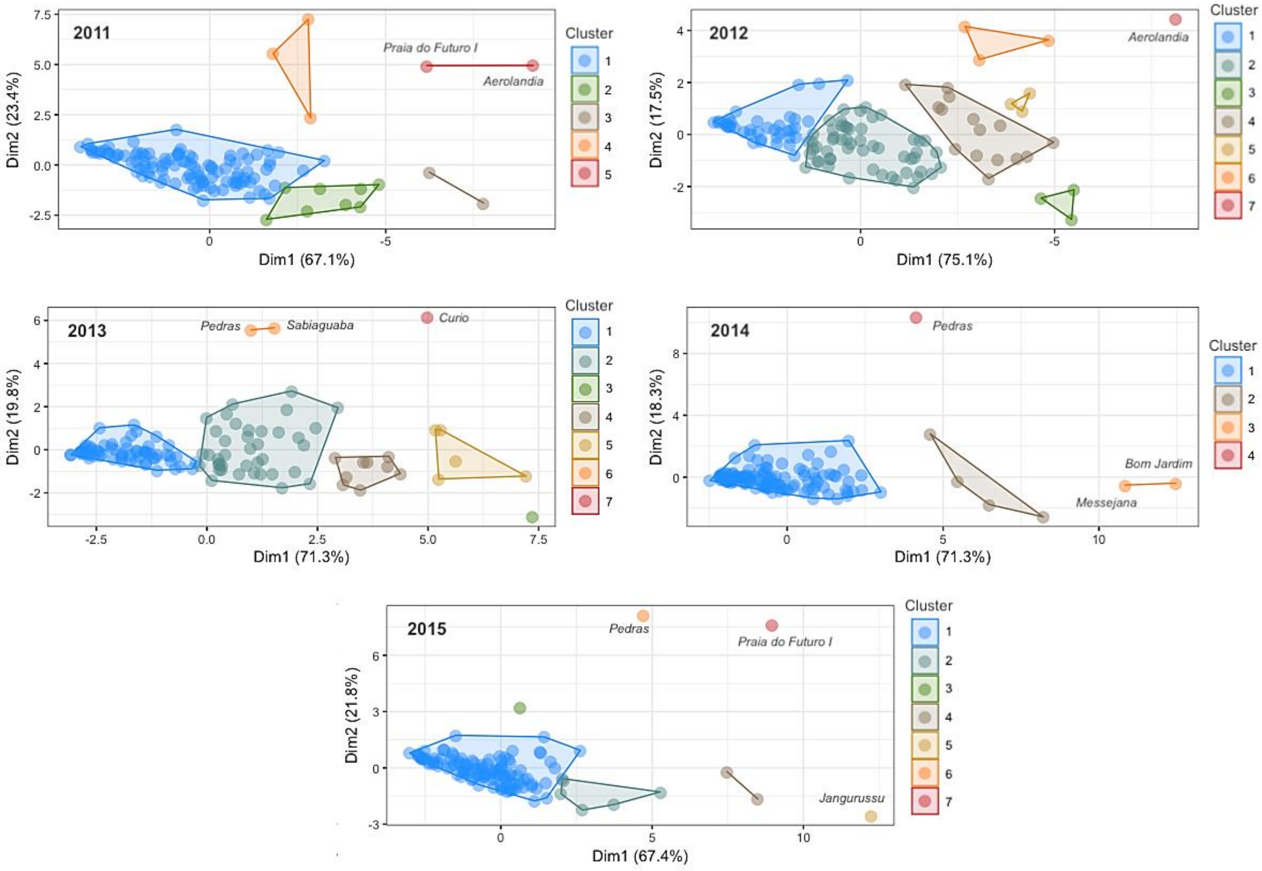

Principal component (PC) plots (S2 Fig) are oriented such that scores on PC1 increase left to right on the x-axis and PC2 scores increase bottom to top on the y-axis. Proportion of variance explained by each PC is listed alongside the axis. PC1 (x-axis) and PC2 (y-axis) correlate with overall dengue burden and intensity of transmission, respectively (S3 Table). Position of *bairro* points on the PCA plot reflects the score of that *bairro* observation on those two principal components; for example, observations in the top left quadrant exhibit low scores on PC1 and higher on PC2, indicating lower than average counts of dengue but the occurrence of high intensity (i.e. incidence rate) transmission during at least one week over the course of the year.

Squared loadings of the first two principal components (PC) were calculated from the normalized variables without rotation using the R package ‘psych’ [1] (S3 Table) to facilitate interpretation of PC plots (S2 Fig). The signs on loadings are arbitrary: their meaning is derived from their difference (i.e. positive contrasted with negative) and relative magnitudes [2]. Since the squared correlation coefficients between parameter variables and components equals 1, the squared loadings show the proportion of variance in variables that is explained by the principal components [3].

References

1. Revelle W. psych. 1.8.10 ed. Evanston, Illinois, USA: Northwestern University; 2018.
2. Jolliffe IT, Cadima J. Principal component analysis: a review and recent developments. Philosophical Transactions of the Royal Society A. 2016;374(2065).
3. Abdi H, Williams, L.J. Principal component analysis. Wiley interdisciplinary reviews: computational statistics. 2010;2(4):433-59.
